# Dynamic modulation of enhancer responsiveness by core promoter elements in living *Drosophila* embryos

**DOI:** 10.1093/nar/gkab1177

**Published:** 2021-12-13

**Authors:** Moe Yokoshi, Koji Kawasaki, Manuel Cambón, Takashi Fukaya

**Affiliations:** Laboratory of Transcription Dynamics, Research Center for Biological Visualization, Institute for Quantitative Biosciences, The University of Tokyo, Bunkyo-ku, Tokyo, Japan; Laboratory of Transcription Dynamics, Research Center for Biological Visualization, Institute for Quantitative Biosciences, The University of Tokyo, Bunkyo-ku, Tokyo, Japan; Applied Mathematics Department, University of Granada, Granada, Spain; Laboratory of Transcription Dynamics, Research Center for Biological Visualization, Institute for Quantitative Biosciences, The University of Tokyo, Bunkyo-ku, Tokyo, Japan; Department of Life Sciences, Graduate School of Arts and Sciences, The University of Tokyo, Bunkyo-ku, Tokyo, Japan

## Abstract

Regulatory interactions between enhancers and core promoters are fundamental for the temporal and spatial specificity of gene expression in development. The central role of core promoters is to initiate productive transcription in response to enhancer's activation cues. However, it has not been systematically assessed how individual core promoter elements affect the induction of transcriptional bursting by enhancers. Here, we provide evidence that each core promoter element differentially modulates functional parameters of transcriptional bursting in developing *Drosophila* embryos. Quantitative live imaging analysis revealed that the timing and the continuity of burst induction are common regulatory steps on which core promoter elements impact. We further show that the upstream TATA also affects the burst amplitude. On the other hand, Inr, MTE and DPE mainly contribute to the regulation of the burst frequency. Genome editing analysis of the pair-rule gene *fushi tarazu* revealed that the endogenous TATA and DPE are both essential for its correct expression and function during the establishment of body segments in early embryos. We suggest that core promoter elements serve as a key regulatory module in converting enhancer activity into transcription dynamics during animal development.

## INTRODUCTION

Communication between enhancers and core promoters is critical for the temporal and spatial specificity of gene expression. Enhancers are distal regulatory elements that contain a cluster of binding sites for sequence-specific transcription factors and co-activators, which usually span several hundreds to thousands base pairs (bp) in length. Recent quantitative imaging studies reported that enhancers are responsible for driving transcriptional bursting from their target genes (e.g. 1–3). However, little is known about the role of core promoters in the process. Core promoters are short segments of DNA, which typically range −40 to +40 relative to the +1 transcription start site (TSS). They serve as a docking site for general transcription factors and RNA polymerase II (Pol II) for the assembly of the pre-initiation complex. Importantly, core promoters alone are not sufficient to drive a high level of transcription, but they typically require enhancer's activation cues to facilitate the assembly of active transcription machineries. Thus, core promoters act as a ‘gateway to transcription’, converting enhancer activity into gene expression (reviewed in 4).

Core promoters consist of several sequence motifs located at fixed positions relative to the TSS. The TATA box (TATA) (5) is typically located 25–30 bp upstream of the TSS and directly interacts with TATA-binding protein (TBP), a subunit of the transcription factor IID (TFIID) complex. Initiator (Inr), motif 10 element (MTE) and downstream promoter element (DPE) are sequence motifs downstream of TATA (6–8) and serve as an additional docking site for TFIID via direct interaction with TBP-associated factors (TAFs) (9–13). In addition, core promoters often contain binding-sites for sequence-specific DNA binding proteins such as Zelda and GAGA factor (GAF) in *Drosophila* (14–16). The precise composition of core promoter elements varies substantially among Pol II-transcribed genes (17–19), which is thought to play a key role in determining the level of gene expression by changing the responsiveness to enhancers (20–23). We previously reported that swapping of entire core promoter sequence can largely alter bursting activities (2). However, it remains to be determined how individual core promoter elements modulate temporal dynamics of gene transcription in living multicellular organisms.

Importantly, quantitative measurement of enhancer responsiveness has been technically challenging with traditional bulk approaches because transcriptional output is controlled not only by core promoter sequences, but also by combinatory effects of surrounding regulatory landscapes such as enhancer strength and chromosome topology (reviewed in 24). Moreover, there is no universal core promoter architecture, making it difficult to compare expression profiles of different genes. Here, we developed a live-imaging system that permits unambiguous comparison of the roles of individual elements using a standardized, optimized synthetic core promoter placed under the control of a fixed enhancer. We have systematically analyzed a series of newly produced *Drosophila* MS2 strains that contain a variety of core promoter modifications at different positions. Quantitative image analysis revealed that each core promoter element differentially modulates the functional parameters of transcriptional bursting in developing embryos. Our data suggest that the timing and the continuity of burst induction are common regulatory steps on which core promoter elements impact. We also show that core promoters use the upstream TATA to increase the amplitude of transcriptional bursting upon activation by distal enhancers. On the other hand, Inr, MTE and DPE mainly contribute to the regulation of the bursting frequency. In addition, promoter-proximal Zelda sites were found to facilitate burst induction at the beginning of new cell cycle, adding another layer of complexity to this process. To further dissect the function of core promoter elements in the context of endogenous genome, we combined genome editing and site-directed transgenesis. Using the pair-rule gene *fushi tarazu* (*ftz*), we show that both TATA and DPE mutations dramatically change transcription dynamics throughout its expression domains, resulting in disrupted spatial patterning of gene expression and misregulation of the downstream segment polarity gene *engrailed* (*en*). We therefore suggest that core promoter elements serve as a key regulatory module in converting enhancer activity into transcription dynamics during animal development.

## MATERIALS AND METHODS

### Experimental model

In all live-imaging experiments, we studied *Drosophila melanogaster* embryos at nuclear cycle 14. The following fly lines were used in this study: *nanos* *>MCP-GFP, His2Av-mRFP/CyO* (25), *DSCP_WT_-MS2-yellow-sna shadow enhancer* (25), *DSCP_mTATA_-MS2-yellow-sna shadow enhancer* (this study), *DSCP_mInr_-MS2-yellow-sna shadow enhancer* (this study), *DSCP_mMTE_-MS2-yellow-sna shadow enhancer* (this study), *DSCP_mDPE_-MS2-yellow-sna shadow enhancer* (this study), *DSCP_mGAGA_-MS2-yellow-sna shadow enhancer* (this study), *DSCP_3xZelda_-MS2-yellow-sna shadow enhancer* (this study), *DSCP_WT_-MS2-yellow-rhoNEE* (this study), *DSCP_mTATA_-MS2-yellow-rhoNEE* (this study), *DSCP_mInr_-MS2-yellow-rhoNEE* (this study), *DSCP_mMTE_-MS2-yellow-rhoNEE* (this study), *DSCP_mDPE_-MS2-yellow-rhoNEE* (this study), *labPr_WT_-MS2-yellow-sna shadow enhancer* (this study), *labPr_TATA_-MS2-yellow-sna shadow enhancer* (this study), *DSCP_WT_-MS2-yellow-gypsy-sna shadow enhancer* (this study), *DSCP_WT_-MS2-yellow No enhancer* (this study), *DSCP_WT_-MS2-yellow-IAB5 enhancer* (this study), *DSCP_mTATA_-MS2-yellow-IAB5 enhancer* (this study), *DSCP_mDPE_-MS2-yellow-IAB5 enhancer* (this study), *snaPr_WT_-MS2-yellow-sna shadow enhancer* (this study), *snaPr_mTATA_-MS2-yellow-sna shadow enhancer* (this study), *DSCP_3xZelda/mTATA_-MS2-yellow-sna shadow enhancer* (this study), *rhoNEE-rhoPr_WT_-MS2-yellow* (this study), *rhoNEE-rhoPr_mTATA_-MS2-yellow* (this study), *rhoNEE-rhoPr_mDPE_-MS2-yellow* (this study), *Δftz-attP/TM6* (this study), *ftz WT core promoter-ftz-HA-24xMS2* (this study), *ftz mTATA core promoter-ftz-HA-24xMS2/TM6* (this study) and *ftz mDPE core promoter-ftz-HA-24xMS2/TM6* (this study).

### Plasmids

Plasmid construction is detailed in the supplementary materials.

### Site-specific transgenesis by phiC31 system

All reporter plasmids were integrated into a unique landing site on the third chromosome using VK00033 strain (26). PhiC31 was maternally provided using *vas-phiC31* strain (27). Microinjection was performed as previously described (28). In brief, 0–1 h embryos were collected and dechorionated with bleach. Aligned embryos were dried with silica gel for ∼7 min and covered with FL-100-1000CS silicone oil (Shin-Etsu Silicone). Subsequently, microinjection was performed using FemtoJet (Eppendorf) and DM IL LED inverted microscope (Leica) equipped with M-152 Micromanipulator (Narishige). Injection mixture typically contains ∼500 ng/μl plasmid DNA, 5 mM KCl, 0.1 mM phosphate buffer, pH 6.8. mini-white marker was used for screening.

### Core promoter modification at endogenous locus

First, endogenous *ftz* transcription unit was removed and replaced with attP site by CRISPR/Cas9-mediated genome editing. Two pCFD3 gRNA expression plasmids and pBS-attP-dsRed donor plasmid were co-injected using *nanos-Cas9/CyO* strain (29). Injection mixture contains 500 ng/μl pCFD3 gRNA expression plasmids, 500 ng/μl pBS-attP-dsRed donor plasmid, 5 mM KCl, 0.1 mM phosphate buffer, pH 6.8. Resulting *ftz* allele was balanced over TM6. Subsequently, *ftz-MS2* plasmid was integrated into the attP site using *Δftz-attP/TM6* strain. Corresponding MS2 plasmid and p3×P3-EGFP.vas-int.NLS plasmid (addgene #60948) were co-injected. Injection mixture contains 500 ng/μl MS2 plasmid, 500 ng/μl phiC31 expression plasmid, 5 mM KCl, 0.1 mM phosphate buffer, pH 6.8. mini-white marker was used for screening. Resulting *ftz* complementation alleles were balanced over TM6. After phiC31-mediated integration, extra sequences derived from plasmid backbone, ampicillin resistant gene and mini-white marker gene were also incorporated into the *ftz* locus.

### cDNA synthesis

Total RNA was extracted from 40 adults of Oregon-R using TRIzol reagent (Thermo Fisher) followed by chloroform purification and isopropanol precipitation. Three μg of total RNA was subjected to reverse transcription using PrimeScript 1st strand cDNA Synthesis Kit (Takara).

### Preparation of probes for *in situ* hybridization

Antisense RNA probes labeled with digoxigenin (DIG RNA Labeling Mix 10× conc., Roche) or biotin (Biotin RNA Labeling Mix 10× conc., Roche) were transcribed using *in vitro* Transcription T7 Kit (Takara). Template DNA for *ftz* probe was PCR amplified from genomic DNA using primers (5′-CGT AAT ACG ACT CAC TAT AGG GTG GGG AAG AGA GTA ACT GAG CAT CGC-3′) and (5′-ATT CGC AAA CTC ACC AGC GT-3′). Template DNA for *en* probe was PCR amplified from cDNA using primers (5′-CGT AAT ACG ACT CAC TAT AGG GCA TGA ACT TGC TTT AGC ACA AAC ATT TCG-3′) and (5′-CAA CTA ATT CAG TCG TTG CGC TCG-3′). Template DNA for *sna* probe was PCR amplified from genomic DNA using primers (5′-CGT AAT ACG ACT CAC TAT AGG GCA GTT GGC TTA ACA GTA CTG-3′) and (5′-ACC TGT CAC AGC CAC CTC AGC-3′). Antisense MS2 probe was *in vitro* transcribed using T3 RNA polymerase (NEB). Templated DNA was prepared by linearizing pBlueScript-MS2 plasmid (30) with EcoRI.

### Fluorescence *in situ* hybridization

Embryos were dechorionated and fixed in fixation buffer (1 ml of 5× PBS, 4 ml of 37% formaldehyde and 5 ml of Heptane) for ∼25 min at room temperature. Vitelline membrane was then removed by shaking embryos in a biphasic mixture of heptane and methanol for ∼1 min. Antisense RNA probes labeled with digoxigenin and biotin were used. Hybridization was performed at 55°C for overnight in hybridization buffer (50% formamide, 5× SSC, 50 μg/ml Heparin, 100 μg/ml salmon sperm DNA, 0.1% Tween-20). Subsequently, embryos were washed with hybridization buffer at 55°C and incubated with Western Blocking Reagent (Roche) at room temperature for 1 h. Then, embryos were incubated with sheep anti-digoxigenin (Roche) and mouse anti-biotin primary antibodies (Invitrogen) at 4°C for overnight, followed by incubation with Alexa Fluor 555 donkey anti-sheep (Invitrogen) and Alexa Flour 488 goat anti-mouse (Invitrogen) fluorescent secondary antibodies at room temperature for 1 h. DNA was stained with DAPI, and embryos were mounted in ProLong Gold Antifade Mountant (Thermo Fisher). Imaging was performed on a Zeiss LSM 900 confocal microscope. Plan-Apochromat 20×/0.8 N.A. objective was used. Images were captured in 16-bit. Maximum projections were obtained for all z-sections, and resulting images were shown. Brightness of images was linearly adjusted using Fiji (https://fiji.sc).

### Cuticle preparation

Eggs were aged ∼24 h at 25.0°C, and dechorionated with bleach. Subsequently, vitelline membrane was removed by shaking in 1:1 methanol: heptane for ∼30 s. Samples were then mounted in 1:1 lactic acid: Hoyer's medium, and incubated at 60°C for overnight. Images were acquired using Optiphot-2 (Nikon) equipped with Moticam 10+ (Motic).

### MS2 live-imaging

**Figure 1.**
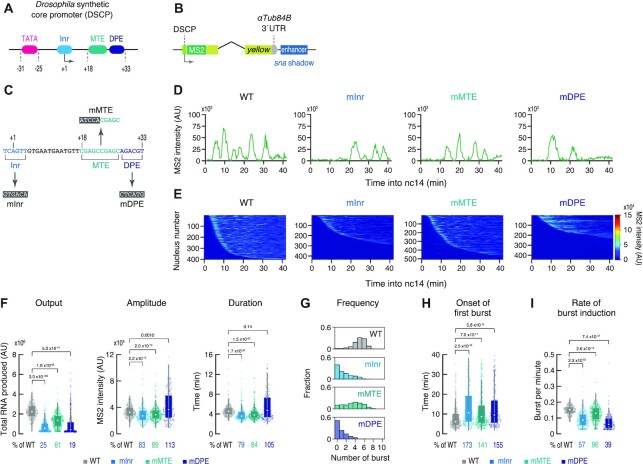
Inr, MTE and DPE mainly control bursting frequency. (**A**) Schematic representation of the *Drosophila* synthetic core promoter (DSCP). (**B**) Schematic representation of the *yellow* reporter gene containing the 155-bp DSCP, the 1.5-kb *sna* shadow enhancer, and 24× MS2 RNA stem loops within the 5′ UTR. (**C**) Inr, MTE and DPE were mutated as indicated. (**D**) Representative trajectories of transcription activity of the MS2 reporter genes with WT (left), mInr (middle left), mMTE (middle right) and mDPE DSCP (right) in individual nuclei. AU; arbitrary unit. (**E**) MS2 trajectories for all analyzed nuclei. Each row represents the MS2 trajectory for a single nucleus. A total of 403, 435, 509 and 444 ventral-most nuclei, respectively, were analyzed from three independent embryos for the reporter genes with WT (left), mInr (middle left), mMTE (middle right) and mDPE DSCP (right). Nuclei were ordered by their onset of transcription in nc14. AU; arbitrary unit. (**F**) Boxplots showing the distribution of total output (left), burst amplitude (middle) and burst duration (right). The box indicates the lower (25%) and upper (75%) quantile and the open circle indicates the median. Whiskers extend to the most extreme, non-outlier data points. A total of 403, 435, 509 and 444 ventral-most nuclei, respectively, were analyzed from three independent embryos for the reporter genes with WT, mInr, mMTE and mDPE DSCP. Median values relative to the WT reporter are shown at the bottom. The *P*-values of two-sided Wilcoxon rank sum test are shown at the top. AU; arbitrary unit. (**G**) Histograms showing the distribution of burst frequency. A total of 403, 435, 509 and 444 ventral-most nuclei, respectively, were analyzed from three independent embryos for the reporter genes with WT (top), mInr (upper middle), mMTE (lower middle) and mDPE DSCP (bottom). (**H**, **I**) Boxplots showing the distribution of the timing of first burst (H) and the burst frequency normalized by the length of time after the first burst (I). The box indicates the lower (25%) and upper (75%) quantile and the open circle indicates the median. Whiskers extend to the most extreme, non-outlier data points. A total of 403, 435, 509 and 444 ventral-most nuclei, respectively, were analyzed from three independent embryos for the reporter genes with WT, mInr, mMTE and mDPE DSCP. Median values relative to the WT reporter are shown at the bottom. The *P*-values of two-sided Wilcoxon rank sum test are shown at the top.

**Figure 2.**
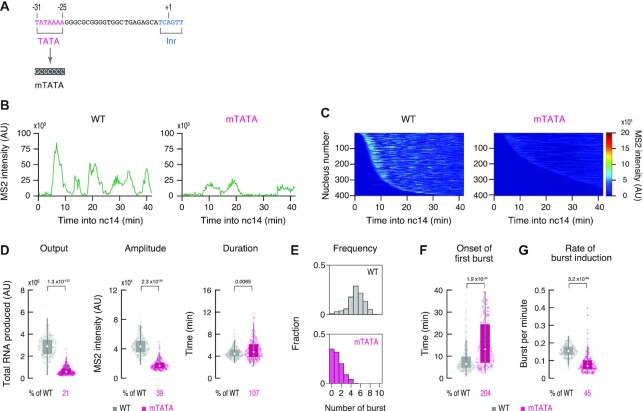
TATA modulates bursting amplitude and frequency. (**A**) TATA was mutated as indicated. Modified core promoter was placed under the control of the *sna* shadow enhancer as illustrated in Figure 1B. (**B**) Representative trajectories of transcription activity of the MS2 reporter genes with WT (left) and mTATA DSCP (right) in individual nuclei. AU; arbitrary unit. (**C**) MS2 trajectories for all analyzed nuclei. Each row represents the MS2 trajectory for a single nucleus. A total of 401 and 406 ventral-most nuclei, respectively, were analyzed from three independent embryos for the reporter genes with WT (left) and mTATA DSCP (right). Nuclei were ordered by their onset of transcription in nc14. AU; arbitrary unit. (**D**) Boxplots showing the distribution of total output (left), burst amplitude (middle) and burst duration (right). The box indicates the lower (25%) and upper (75%) quantile and the open circle indicates the median. Whiskers extend to the most extreme, non-outlier data points. A total of 401 and 406 ventral-most nuclei, respectively, were analyzed from three independent embryos for the reporter genes with WT and mTATA DSCP. Median values relative to the WT reporter are shown at the bottom. The *P*-values of two-sided Wilcoxon rank sum test are shown at the top. AU; arbitrary unit. (**E**) Histograms showing the distribution of burst frequency. A total of 401 and 406 ventral-most nuclei, respectively, were analyzed from three independent embryos for the reporter genes with WT (top) and mTATA DSCP (bottom). (**F**, **G**) Boxplots showing the distribution of the timing of first burst (F) and the burst frequency normalized by the length of time after the first burst (G). The box indicates the lower (25%) and upper (75%) quantile and the open circle indicates the median. Whiskers extend to the most extreme, non-outlier data points. A total of 401 and 406 ventral-most nuclei, respectively, were analyzed from three independent embryos for the reporter genes with WT and mTATA DSCP. Median values relative to the WT reporter are shown at the bottom. The *P*-values of two-sided Wilcoxon rank sum test are shown at the top.

**Figure 3.**
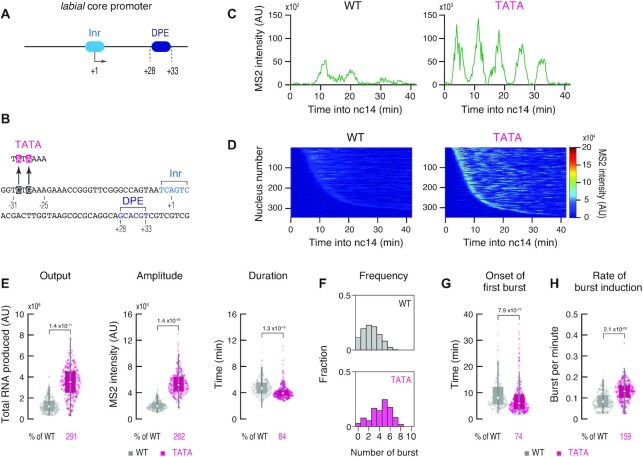
Engineering of an endogenous TATA-less core promoter. (**A**) Endogenous *lab* core promoter contains Inr and DPE, but lacks TATA. The *sna* shadow enhancer was used for the analysis. (**B**) Two nucleotides substitution was introduced to contain an optimal TATA. (**C**) Representative trajectories of transcription activity of the MS2 reporter genes with unmodified (left) and modified *lab* core promoter (right) in individual nuclei. AU; arbitrary unit. (**D**) MS2 trajectories for all analyzed nuclei. Each row represents the MS2 trajectory for a single nucleus. A total of 344 and 348 ventral-most nuclei, respectively, were analyzed from three independent embryos for the reporter genes with unmodified (left) and modified *lab* core promoter (right). Nuclei were ordered by their onset of transcription in nc14. AU; arbitrary unit. (**E**) Boxplots showing the distribution of total output (left), burst amplitude (middle) and burst duration (right). The box indicates the lower (25%) and upper (75%) quantile and the open circle indicates the median. Whiskers extend to the most extreme, non-outlier data points. A total of 344 and 348 ventral-most nuclei, respectively, were analyzed from three independent embryos for the reporter genes with unmodified and modified *lab* core promoter. Median values relative to the unmodified *lab* core promoter are shown at the bottom. The *P*-values of two-sided Wilcoxon rank sum test are shown at the top. AU; arbitrary unit. (**F**) Histograms showing the distribution of burst frequency. A total of 344 and 348 ventral-most nuclei, respectively, were analyzed from three independent embryos for the reporter genes with unmodified (top) and modified *lab* core promoter (bottom). (**G**, **H**) Boxplots showing the distribution of the timing of first burst (G) and the burst frequency normalized by the length of time after the first burst (H). The box indicates the lower (25%) and upper (75%) quantile and the open circle indicates the median. Whiskers extend to the most extreme, non-outlier data points. A total of 344 and 348 ventral-most nuclei, respectively, were analyzed from three independent embryos for the reporter genes with unmodified and modified *lab* core promoter. Median values relative to the unmodified *lab* core promoter are shown at the bottom. The *P*-values of two-sided Wilcoxon rank sum test are shown at the top.

**Figure 4.**
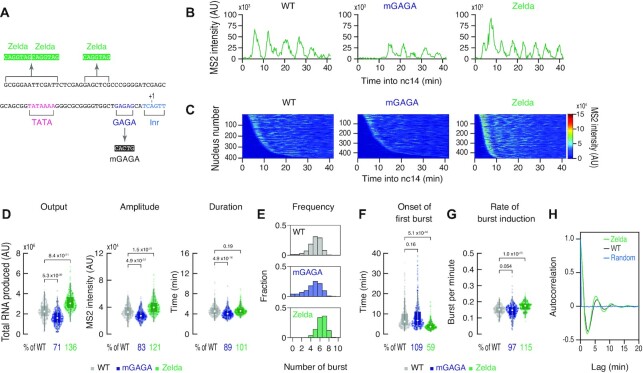
Zelda facilitates rapid induction of transcriptional bursting. (**A**) DSCP was modified to mutate GAGA site or add three optimal Zelda binding sites. The *sna* shadow enhancer was used for the analysis. (**B**) Representative trajectories of transcription activity of the MS2 reporter genes with WT (left), mGAGA (middle) and Zelda DSCP (right) in individual nuclei. AU; arbitrary unit. (**C**) MS2 trajectories for all analyzed nuclei. Each row represents the MS2 trajectory for a single nucleus. A total of 403, 458 and 458 most ventral-nuclei, respectively, were analyzed from three individual embryos for the reporter genes with WT (left), mGAGA (middle) and Zelda DSCP (right). Nuclei were ordered by their onset of transcription in nc14. Panel of WT is the same as the panel in Figure 1E. AU; arbitrary unit. (**D**) Boxplots showing the distribution of total output (left), burst amplitude (middle) and burst duration (right). The box indicates the lower (25%) and upper (75%) quantile and the open circle indicates the median. Whiskers extend to the most extreme, non-outlier data points. A total of 403, 458 and 458 most ventral-nuclei, respectively, were analyzed from three independent embryos for the reporter genes with WT, mGAGA and Zelda DSCP. Median values relative to the WT DSCP reporter are shown at the bottom. The *P*-values of two-sided Wilcoxon rank sum test are shown at the top. Plot of WT is the same as the plot in Figure 1F. AU; arbitrary unit. (**E**) Histograms showing the distribution of burst frequency. A total of 403, 458 and 458 ventral-most nuclei, respectively, were analyzed from three independent embryos for the reporter genes with WT (top), mGAGA (middle) and Zelda DSCP (bottom). Plot of WT is the same as the plot in Figure 1G. (**F**, **G**) Boxplots showing the distribution of the timing of first burst (F) and the burst frequency normalized by the length of time after the first burst (G). The box indicates the lower (25%) and upper (75%) quantile and the open circle indicates the median. Whiskers extend to the most extreme, non-outlier data points. A total of 403, 458 and 458 ventral-most nuclei, respectively, were analyzed from three independent embryos for the reporter genes with WT, mGAGA and Zelda DSCP. Median values relative to the WT reporter are shown at the bottom. The *P*-values of two-sided Wilcoxon rank sum test are shown at the top. Plot of WT is the same as the plot in Figure 1H and I. (**H**) Autocorrelation analysis of MS2 trajectories of the reporter genes with WT and Zelda DSCP. A total of 403 and 458 most ventral nuclei, respectively, were analyzed. Each trace indicates mean autocorrelation value from all analyzed nuclei. Random represents mean autocorrelation value calculated from time-shuffled MS2 trajectories of the WT reporter. Autocorrelation values were calculated with 16.8 s timestep and normalized to 1 at the smallest lag time.

**Figure 5.**
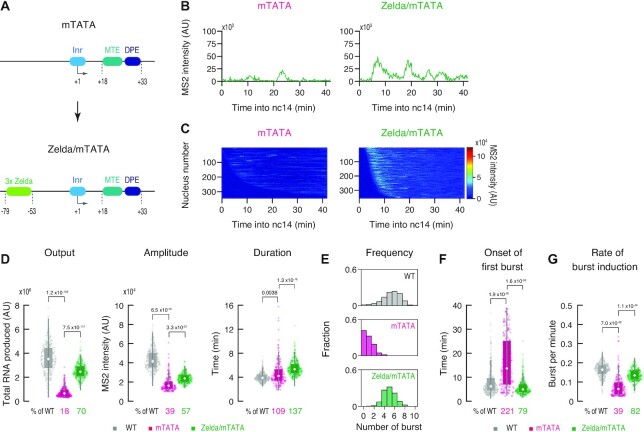
Zelda compensates weak activity of TATA-less core promoters. (**A**) Schematic representation of mTATA and Zelda/mTATA DSCP. The *sna* shadow enhancer was used for the analysis. (**B**) Representative trajectories of transcription activity of the MS2 reporter genes with mTATA (left) and Zelda/mTATA DSCP (right) in individual nuclei. AU; arbitrary unit. (**C**) MS2 trajectories for all analyzed nuclei. Each row represents the MS2 trajectory for a single nucleus. A total of 343 and 359 ventral-most nuclei, respectively, were analyzed from three independent embryos for the reporter genes with mTATA (left) and Zelda/mTATA DSCP (right). Nuclei were ordered by their onset of transcription in nc14. AU; arbitrary unit. (**D**) Boxplots showing the distribution of total output (left), burst amplitude (middle) and burst duration (right). The box indicates the lower (25%) and upper (75%) quantile and the open circle indicates the median. Whiskers extend to the most extreme, non-outlier data points. A total of 371, 343 and 359 ventral-most nuclei, respectively, were analyzed from three independent embryos for the reporter genes with WT, mTATA and Zelda/mTATA DSCP. Median values relative to the WT reporter are shown at the bottom. The *P*-values of two-sided Wilcoxon rank sum test are shown at the top. (**E**) Histograms showing the distribution of burst frequency. A total of 371, 343 and 359 ventral-most nuclei, respectively, were analyzed from three independent embryos for the reporter genes with WT (top), mTATA (middle) and Zelda/mTATA DSCP (bottom). (**F**, **G**) Boxplots showing the distribution of the timing of first burst (F) and the burst frequency normalized by the length of time after the first burst (G). The box indicates the lower (25%) and upper (75%) quantile and the open circle indicates the median. Whiskers extend to the most extreme, non-outlier data points. A total of 371, 343 and 359 ventral-most nuclei, respectively, were analyzed from three independent embryos for the reporter genes with WT, mTATA and Zelda/mTATA DSCP. Median values relative to the WT reporter are shown at the bottom. The *P*-values of two-sided Wilcoxon rank sum test are shown at the top.

Virgin females of *nanos* *>MCP-GFP, His2Av-mRFP/CyO* (25) were mated with males carrying the MS2 allele. The resulting embryos were dechorionated and mounted between a polyethylene membrane (Ube Film) and a coverslip (18 mm × 18 mm), and embedded in FL-100–450CS (Shin-Etsu Silicone). Embryos were imaged using a Zeiss LSM 800 (Figures 1 and 4) or LSM 900 (Figures 2, 3, 5, 7, Supplementary Figures S1, S3, S5, S6, S8 and S10). Temperature was kept in between 23.5 and 25.0°C during imaging. Plan-Apochromat 40×/1.4 N.A. oil immersion objective was used. In Figures 1, 2, 4, 7 and Supplementary Figure S5, a stack of 26 images separated by 0.5 μm was acquired at each time point, and the final time resolution is 16.8 s/frame. In Figures 3, 5, Supplementary Figures S1, S3, S6, S8 and S10, a stack of 20 images separated by 0.63 μm was acquired at each time point, and the final time resolution is 12.8 s/frame. Images were captured in 16-bit. Images were typically taken from the end of nc13 to the onset of gastrulation at nc14. During imaging, data acquisition was occasionally stopped for a few seconds to correct z-position, and data were concatenated afterwards. For each cross, three biological replicates were taken. The same laser power and microscope setting were used for each set of experiments.

### Image analysis

All the image processing methods and analysis were implemented in MATLAB (R2019a, R2019b MathWorks).

### Segmentation of nuclei

For each time point, maximum projection was obtained for all z-sections per image. His2Av-mRFP was used to segment nuclei. 512 × 512 maximum projection images were initially cropped into 430 × 430 (Figures 1, 4, 7) or 300 × 430 (Figures 2, 3, 5, Supplementary Figures S1, S3, S8 and S10) to remove nuclei at the edge, and used for subsequent analysis. In the analysis of *rho*NEE reporters (Supplementary Figures S5 and S6), 512 × 512 maximum projection images were initially cropped into 300 × 500 to remove nuclei outside of the expression domain. For nuclei segmentation, we used two different methods. In the first method, His2Av images were pre-processed with Gaussian filtering, top-hap filtering, and adaptive histogram equalization, in order to enhance the signal-to-noise contrast. Processed images were converted into binary images using a threshold value obtained from Otsu's method. Nuclei were then watershedded to further separate and distinguish from neighboring nuclei. Subsequently, binary images were manually corrected using Fiji (https://fiji.sc). In the second method, His2Av images were first blurred with Gaussian filter to generate smooth images. Pixels expressing intensity higher than 5% of the global maxima in the histogram of His2Av channel were removed. Processed images were converted into binary images using a custom threshold-adaptative segmentation algorithm. Threshold values were determined at each time frame by taking account of (i) the histogram distribution of His2Av channel and (ii) the number and the size of resulting connected components. Boundaries of components were then modified to locate MS2 transcription dots inside of nearest nuclei. In brief, pixels with intensity twice larger than mean intensity of MS2 channel were considered as transcription dots, and new binary images were created for each time frame. The Euclidean distances between the centroid of binarized transcription dot and all boundaries of segmented nuclei were calculated. Boundary of the nucleus with the smallest Euclidean distance was modified in order to capture transcription dot within a nucleus. Centroids of connected components in nuclei segmentation channel were used to compute the Voronoi cells of the image. Resulting binary images were manually corrected by using Fiji (https://fiji.sc).

### Tracking of nuclei

Nuclei tracking was done by finding the object with minimal movement across the frames of interest. For each nucleus in a given frame, the Euclidean distances between the centroids of the nucleus in the current time frame and the nuclei in the next or previous time frame were determined. The nucleus with the minimum Euclidean distance was considered as the same lineage.

### Recording of MS2 signals

3D raw images with all z-sections of MCP-GFP channel were used to record MS2 fluorescence signals. Using segmented regions from max projected images of His2Av-mRFP channel, fluorescence intensities within each nucleus were extracted. 3D fluorescence values were assigned to the nearest segmented regions of projected images. Signals of MS2 transcription dots were determined by calculating an integral of fluorescence intensities around the brightest pixel within each nucleus using a 2D Gaussian fitting method as described below. (i) The xyz position of transcription site was determined as a brightest pixel in each nucleus. (ii) A 2D Gaussian fitting was performed in a 11 × 11 pixels region with a single z-plane centering the transcription site to estimate a fluorescent dot intensity and local background. Fitting was performed with the following formula

$$\begin{equation*}I\ \left( {x,y} \right) = \ \alpha + {I_{0\ }}{{\rm exp}^{\left( { - \left( {\frac{{{{\left( {x - {x_0}} \right)}^2}}}{{2{\sigma _x}^2}} + \frac{{{{\left( {y - {y_0}} \right)}^2}}}{{2{\sigma _y}^2}}} \right)} \right)}}\end{equation*}$$

where *α* is the local background intensity, *I*_0_ is the amplitude of the peak fluorescence intensity, *x*_0_ and *y*_0_ are the center of the peak, *σ_x_* and *σ_y_* are the spreads of the fluorescent dot. When the size of estimated fluorescent dot was larger than a fitted region of 11 × 11 pixels due to a low-quality fitting, whole time-point data of corresponding nucleus was excluded from further analysis. (iii) The intensity of MS2 transcription dot was calculated as $2\pi {\sigma _x}{\sigma _y}{I_0}$ from fitting parameters as an estimated integral value after subtracting the local background (31). Subsequently, minimum MS2 intensities were determined for individual trajectories and subtracted to make the baseline zero.

### Detection of transcriptional bursting

A transcriptional burst was defined as a local change in fluorescence intensity. First, signal trajectories were smoothed by averaging within a window of 5 timeframes. When a nucleus had above-threshold transcription activity, burst was considered to be started. Burst was considered to be ended when the intensity dropped below 55% of the local peak value of each burst. Location of defined burst was then moved 2 timeframes afterwards to better capture the center of individual bursting event. When the burst duration is less than 5 timeframes, it was considered as a false-positive derived from detection noise. It was confirmed that this filtering method does not affect quantification of the burst duration (Supplementary Figure S4E and F). When signal trace exhibited continuous decreasing at the beginning of burst detection, it was also not considered as a burst. Same method and threshold value were used for each set of experiments.

### Description of bursting properties

From each trajectory, number of bursts, amplitude and duration of each burst, and total integrated signal (output) produced by each nucleus were measured. To determine amplitude, the peak value during the burst was measured using trajectories after smoothing by averaging within a window of 5 timeframes. The duration was determined by measuring the length of each burst. Total output was measured by taking the area under the raw trajectory. The amplitude and duration for each nucleus were determined by taking average of all analyzed bursts in a single nucleus. The rate of burst induction was determined as a frequency divided by a length of time after the initial burst for each active nucleus.

### Fraction of instantaneously and cumulative active nuclei

For each time frame, nuclei with MS2 intensity above the threshold were considered as active. Threshold was determined for each group of nuclei by calculating the 10% of the maximum MS2 intensity across all trajectories.

### Mean MS2 intensity per actively transcribing nucleus

MS2 intensities in instantaneously active nuclei at each time point were averaged.

### Autocorrelation analysis of MS2 signal

Autocorrelation values were used to estimate a periodicity of each MS2 trajectory. First, MS2 trajectories were smoothed as described in the previous section. To extract periodicity, the first derivative of smoothed MS2 signal was calculated to exclude general trends of signal changes during nc14. The autocorrelation value was calculated with 16.8 s timestep lags according to the time resolution of live-imaging data (16.8 s/frame). All autocorrelation values were normalized to 1 at the smallest lag time. Random data was generated from time-shuffled MS2 trajectories of the WT reporter by using MATLAB rand function.

### Computational reconstitution of *ftz* expression

Newly synthesized *ftz* mRNAs were considered to be linearly degraded with a half-life of 7 min according to previous measurements in early embryos (32). Amount of *ftz* mRNA remained to be undegraded by the end of analysis was estimated using live-imaging data in Figure 7. Using segmentation mask, individual nuclei were false-colored with the pixel intensity proportional to the level of intact *ftz* mRNA in a given nucleus. Resulting image was then colored and layered over the maximum projected image of His2Av-mRFP.

## RESULTS

### Inr, MTE and DPE modulate bursting frequency

To quantitatively visualize how each core promoter element regulates enhancer responsiveness in living *Drosophila* embryos, we employed the MS2/MCP live-imaging method (33,34). We constructed a reporter system in which the *yellow* gene was placed under the control of the *Drosophila* synthetic core promoter (DSCP) (35), a modified *even-skipped* (*eve*) core promoter containing TATA, Inr, MTE and DPE at optimal positions relative to the TSS (Figure 1A, Supplementary Figure S1A). The use of a standardized core promoter architecture permits systematic comparison of individual elements in an unambiguous manner. A 24× MS2 RNA stem-loop sequence was engineered into the 5′ untranslated region (UTR) to enable visualization of nascent RNA production with a maternally provided MCP-GFP fusion protein. *DSCP-MS2-yellow* reporter gene was placed under the control of a full-length 1.5-kb *snail* (*sna*) shadow enhancer (Figure 1B), which drives expression in the ventral region of early embryos (Supplementary Figure S1C) (36,37). It has been previously shown that the *sna* shadow enhancer, but not the proximal primary enhancer, is essential for the mesoderm invagination during gastrulation (36,37). Thus, the use of the *sna* shadow enhancer provides a nice model for visualizing transcriptional regulation by developmentally essential enhancers. In this synthetic locus, *sna* shadow enhancer is located ∼6.5 kb away from the TSS, which is similar to the enhancer-promoter distance found at the endogenous *sna* locus (∼7 kb). Transgenes were integrated into a specific genomic landing site via phiC31-mediated transgenesis (26,38). Importantly, expression of the reporter gene was almost completely abolished upon deletion of enhancer sequence from the synthetic locus (Supplementary Figure S1B–E), indicating that the induction of transcription from DSCP is mediated by a linked enhancer.

First, we examined the core promoter elements Inr, MTE and DPE by introducing mutations that compromise their direct interaction with TFIID (Figure 1C) (6,7,9,13,39). Notably, the mutation at the +18 to +22 positions eliminates MTE-dependent transcription without affecting DPE function (7,13), allowing us to examine the individual contributions of MTE and DPE in the same core promoter context. Transcription activity was monitored from the entry into nuclear cycle 14 (nc14), when the major wave of zygotic genome activation starts to take place. To unambiguously compare the activities of different reporter genes at the same dorsal-ventral (DV) position, we focused on the ventral-most nuclei, as defined by the location of ventral furrow formation at the onset of gastrulation (Supplementary Figure S2A). MS2 signal intensity and background fluorescence were determined by performing a 2D Gaussian fit at the z-plane corresponding to the highest intensity value for each time point (Supplementary Figure S2B and C). Quantitative image analysis revealed that the *sna* shadow enhancer induces fewer number of transcriptional bursts from the MTE mutant (mMTE) than the WT (Figure 1D, Supplementary Movie S1). In comparison, even less frequent bursts were observed when the Inr mutant (mInr) and the DPE mutant (mDPE) were linked to the enhancer (Figure 1D, Supplementary Movie S1). This trend was clearly seen when MS2 trajectories in all analyzed nuclei were visualized as a heatmap (Figure 1E). These data suggest that each of these elements individually contributes to the regulation of transcriptional bursting, with Inr/DPE playing a major role and MTE playing a more supplemental role. Similar reduction of burst induction was also seen when enhancer-promoter communication was attenuated by the insertion of *gypsy* insulator (40) (Supplementary Figure S3), supporting the idea that inefficient TFIID recruitment to the core promoter region reduces responsiveness to enhancers. We then analyzed individual bursting events and quantified their functional parameters, including amplitude, duration, and frequency (Supplementary Figure S2D). As seen in individual MS2 trajectories (Figure 1D), the frequency of transcriptional bursting and the total output were largely diminished when each of these elements was mutated (Figure 1F and G, Supplementary Figure S4A). On the other hand, the amplitude and the duration remained to be less affected (Figure 1F, Supplementary Figure S4E and F), although Inr and MTE mutations caused moderate yet significant reduction in these parameters (Figure 1F). It should be also noted that DPE mutation increased variance of the duration and the amplitude within a population (Figure 1F), implicating that the direct interaction between TFIID and DPE helps to ensure production of homogeneous transcriptional bursts. Importantly, all core promoter variants exhibited significant delay in the onset of the first round of transcription (Figure 1H), and substantial fraction of nuclei remained inactive during the analysis even though the *sna* shadow enhancer itself is in an active state as evidenced by the profile of the WT reporter (Supplementary Figure S4B). We next determined the rate of burst induction as a number of bursts divided by a time length after the onset of first burst for each active nucleus. There was an overall reduction in the efficiency of producing subsequent bursts after the first round of burst in all core promoter variants (Figure 1I). These data suggest that reduced bursting frequency is attributed to (i) the initial delay of first burst and (ii) the inefficient induction of subsequent bursts in response to activation cues from distal enhancers. Essentially same results were observed when the *sna* shadow enhancer was replaced with another key developmental enhancer, *rhomboid* neuroectoderm element (*rho*NEE) (41) (Supplementary Figure S5). Furthermore, reduction of the bursting frequency was also seen when DPE was mutated from the minimal *rho* core promoter linked to its cognate *rho*NEE enhancer placed in a same distance as in the endogenous locus (Supplementary Figure S6). Overall, our data suggest that Inr, MTE and DPE support rapid and consecutive induction of transcriptional bursting, thereby facilitating the production of a high level of nascent transcripts during early development. Lastly, to obtain kinetic information underlying burst regulation, we examined the distribution of ON and OFF duration of individual events (Supplementary Figure S7). We found that OFF duration between two consecutive bursts nicely fits to a single exponential distribution, suggesting that burst initiation is driven by a single rate-limiting step. On the other hand, ON duration of individual bursts did not fit well to an exponential distribution, implicating that multi-step kinetics are involved to turn the promoter off.

### TATA modulates bursting frequency and amplitude

We next examined the role of the upstream TATA, which we mutated according to previous characterization in order to abrogate its function (Figure 2A) (7,21). Live-imaging analysis revealed that the TATA mutation causes more profound changes in overall transcription activity comparing to other modifications (Figure 2B-E, Supplementary Figure S4C, Supplementary Movie S2). Importantly, the *sna* shadow enhancer could only induce bursts with substantially lower amplitude from the TATA mutant (mTATA) (Figure 2D), suggesting that TATA mutation largely diminishes the number of Pol II released per burst. These results are consistent with previous studies showing that TATA affects the burst size and gene expression noise in yeast and mammalian systems (3,42–48). Burst duration was largely unaffected by the loss of TATA (Figure 2D, Supplementary Figure S4F), indicating that the size of each burst is mainly determined by the amplitude, but not the duration, of individual activation events. There also was a clear reduction in the frequency of transcriptional bursting when TATA was mutated (Figure 2E). As seen for other modifications (Figure 1H and I), reduced frequency appears to be attributed to delayed and inefficient burst induction (Figure 2F and G, Supplementary Figure S4D). Strong TATA-dependency was also observed when DSCP was placed under the control of the *rho*NEE (Supplementary Figure S5) or the minimal *sna* and *rho* core promoters were linked to their cognate enhancers (Supplementary Figures S6 and S8). We therefore suggest that core promoters use the upstream TATA to ensure rapid and consecutive induction of strong bursts upon activation by enhancers.

We then tested if the conversion of a natural TATA-less core promoter into a TATA-containing core promoter has an opposite effect. It has been previously shown that the core promoter regions of the *Drosophila* homeotic (Hox) genes are typically depleted of TATA (49). Indeed, the core promoter of the Hox gene *labial* (*lab*) lacks an optimal TATA motif (Figure 3A and B). When the *lab* core promoter was placed under the control of the *sna* shadow enhancer, only weak and infrequent bursts were produced (Figure 3C and D). We then introduced two nucleotides substitution at the −28 and −30 positions to covert ‘TCTGAAA’ to an optimal TATA motif, ‘TATAAAA’ (Figure 3B). Intriguingly, this minimal modification dramatically increased overall activities including the amplitude and the total output of transcriptional bursting (Figure 3C–F, Supplementary Movie S3). In addition, the timing of first burst (Figure 3G, Supplementary Figure S9A) and the continuity of subsequent bursts (Figure 3H) were also augmented, resulting in a higher frequency of transcriptional bursting (Figure 3F). These results support the idea that the upstream TATA helps to facilitate consecutive induction of strong transcriptional bursting. Burst duration was only moderately impacted by this modification (Figure 3E), implicating that TATA does not largely alter the stability of active transcription machineries at the core promoter region. We next examined if enhancers regulating the expression of natural TATA-less genes also exhibit strong TATA-dependency. To this end, a well-characterized IAB5 enhancer that regulates the expression of the TATA-less Hox gene *Abdominal-B* (*Abd-B*) was linked to the mTATA DSCP (50) (Supplementary Figure S10A). Similar to the *sna* shadow and *rho*NEE enhancers (Figure 2, Supplementary Figure S5), there was a clear reduction in the amplitude and the frequency of transcriptional bursting (Supplementary Figure S10B-G), suggesting that IAB5 enhancer can also utilize TATA to facilitate induction of strong bursts. On the other hand, DPE mutation was found to preferentially impact the bursting frequency (Supplementary Figure S10B-G). These data support the idea that TATA and DPE can differentially modulate bursting profiles under various genomic configurations.

### Promoter-proximal Zelda sites facilitate burst induction

In *Drosophila*, core promoters often contain binding sites for sequence-specific DNA binding proteins, Zelda and GAF (15,51), that are thought to recruit chromatin remodeling complexes such as NURF to increase chromatin accessibility of regulatory regions (52–54). However, it remains unclear how they modulate core promoter functions because previous studies have mainly focused on their roles at enhancer regions (e.g. 30,53,55,56). DSCP contains a GAF-binding site (GAGA) from the *eve* core promoter (Supplementary Figure S1A) (57), but lacks any known Zelda binding motifs. To examine how promoter-proximal GAGA and Zelda sites influence transcription dynamics, we either mutagenized GAGA site or added Zelda sites (Figure 4A). We found that the GAGA mutant (mGAGA) exhibits only a moderate reduction in the bursting amplitude and duration (Figure 4B–D). On the other hand, addition of Zelda sites led to rapid induction of strong bursts (Figure 4B and C, Supplementary Figure S9B) and an overall increase in transcription activities (Figure 4D and E, Supplementary Movie S4). Especially, the timing of initial burst was found to be dramatically accelerated in the presence of Zelda sites (Figure 4F, Supplementary Figure S9B), suggesting that Zelda helps to increase the enhancer responsiveness by opening up the promoter chromatin to allow subsequent TFIID recruitment during the onset of nc14. On the other hand, addition of Zelda sites only moderately increased the continuity of burst induction after the first round of transcription (Figure 4G). Intriguingly, we noticed that periodic patterns of burst induction are present in the heatmap profile of the Zelda reporter gene (Figure 4C). To examine if the addition of Zelda sites actually facilitates to drive periodic transcription, we detected repeating patterns in MS2 trajectories by performing autocorrelation analysis. Although the unmodified WT reporter also drives periodic bursts, our data showed that the Zelda reporter gene exhibits even stronger periodicity (Figure 4H, Supplementary Figure S11). At this point, molecular mechanism underlying periodic bursts is unclear, but these data suggest that it typically takes ∼5–6 min for core promoters to be ready for responding to next activation cue from distal enhancers once Pol II is released for transcriptional elongation in this system.

### Zelda can compensate weak activities of TATA-less core promoters

It is known that key segmentation genes such as *hairy* and *paired* lack TATA but contain promoter-proximal Zelda sites in *Drosophila* (58). To dissect the role of Zelda in the context of TATA-less core promoters, Zelda sites were added to the mTATA DSCP reporter (Figure 5A). This modification led to partial increase in the amplitude and the duration (Figure 5B–D, Supplementary Movie S5). In addition, this reporter gene exhibited substantial increase in the frequency of transcriptional bursting (Figure 5E). Intriguingly, the onset of first burst from the Zelda/mTATA reporter became even earlier than the unmodified WT reporter (Figure 5F), suggesting that Zelda can facilitate the recruitment of TFIID to the core promoter region even in the absence of TATA. The rate of burst induction also became ∼2-fold higher upon addition of Zelda sites (Figure 5G). When the same modification was introduced to the WT DSCP, the rate of burst induction was only moderately increased (Figure 4G), implicating that the burst induction rate was already saturated when all the other elements were present. Overall, our data suggest that the promoter opening by Zelda can compensate low enhancer responsiveness of TATA-less core promoters to facilitate rapid and consecutive induction of transcriptional bursting.

### Core promoter modification at the endogenous *ftz* locus

**Figure 6.**
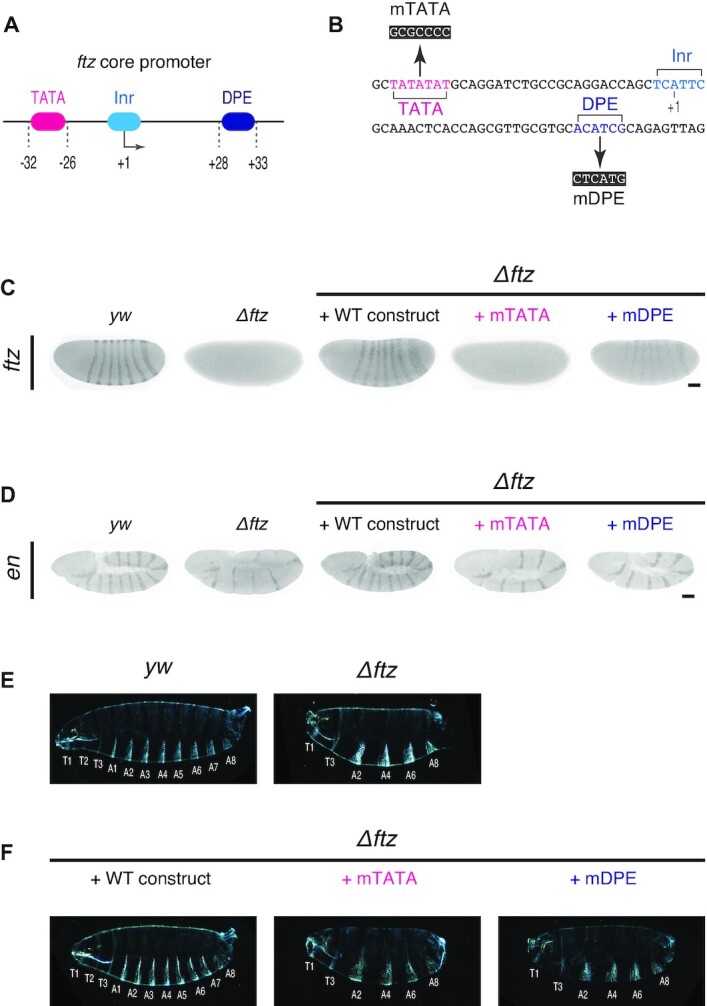
TATA and DPE are both required for proper *ftz* expression and function. (**A**) *ftz* core promoter contains TATA, Inr and DPE. (**B**) TATA and DPE were mutated as indicated. (**C**) Fluorescent *in situ* hybridization of *ftz*. Embryos at late nc14 are shown. *yw* embryo is shown as a control. *ftz-MS2* constructs were integrated into the attP site at the *Δftz* allele. Images are cropped and rotated to align embryos (anterior to the left and posterior to the right). Scale bar indicates 50 μm. (**D**) Fluorescent *in situ* hybridization of *en*. Embryos after germband extension are shown. *yw* embryo is shown as a control. Images are cropped and rotated to align embryos (anterior to the left and posterior to the right). Brightness of each embryo was differentially adjusted for visualization of *en* expression pattern. Scale bar indicates 50 μm. (**E**) Dark-field micrographs showing cuticle preparations of *yw* (left) and *Δftz* (right) larvae. (**F**) Dark-field micrographs showing cuticle preparations of *Δftz* larvae containing WT (left), mTATA (middle) and mDPE *ftz-MS2* transgene (right).

To explore how core promoter elements impact enhancer-promoter communication at the endogenous locus, we next focused on one of the best studied developmental patterning genes, *fushi tarazu* (*ftz*) (59–63). *ftz* is expressed in seven transverse stripes spanning across the anterior-posterior (AP) axis and is regulated by multiple enhancers located 5′ and 3′ of the transcription unit (64–68). The *ftz* core promoter contains TATA, Inr and DPE elements (Figure 6A) (49). Reporter assays in *Drosophila* S2 cultured cells have suggested that DPE, but not TATA, is specifically required for the activation of the *ftz* core promoter by the homeodomain-containing transcription factor Caudal (49). In this model, mutation of DPE is expected to most severely affect *ftz* expression in stripe 5 and 6 because they are regulated by Caudal-dependent enhancers (69). To test this idea, we developed a genome engineering approach for visualizing impacts of core promoter modification at the endogenous locus. First, the entire *ftz* transcription unit was replaced with attP site via CRISPR/Cas9-mediated genome editing (Supplementary Figure S12A). Subsequently, a full-length *ftz* transcription unit containing the modified *ftz* core promoter and 24× MS2 sequence was integrated into the attP site via phiC31-mediated transgenesis (Figure 6B, Supplementary Figure S12A). Fluorescent *in situ* hybridization assay revealed that *ftz* expression is diminished equally across all seven stripes upon DPE mutation (Figure 6C), indicating that the *ftz* core promoter uses DPE for responding not only to Caudal-dependent stripe 5 and 6 enhancers but also to all the other stripe enhancers. Moreover, *ftz* expression was almost completely lost upon TATA mutation (Figure 6C). As a consequence of irregular *ftz* patterning, expression of the downstream segment polarity gene *engrailed* (*en*) was specifically lost from even-numbered stripes in mTATA and mDPE embryos (Figure 6D). Cuticle preparation analysis further revealed that the misexpression of *ftz* and *en* leads to loss of even-numbered body segments in developing embryos (Figure 6E and F). We therefore suggest that both TATA and DPE are required for the correct expression and the function of *ftz* during *Drosophila* embryogenesis.

**Figure 7.**
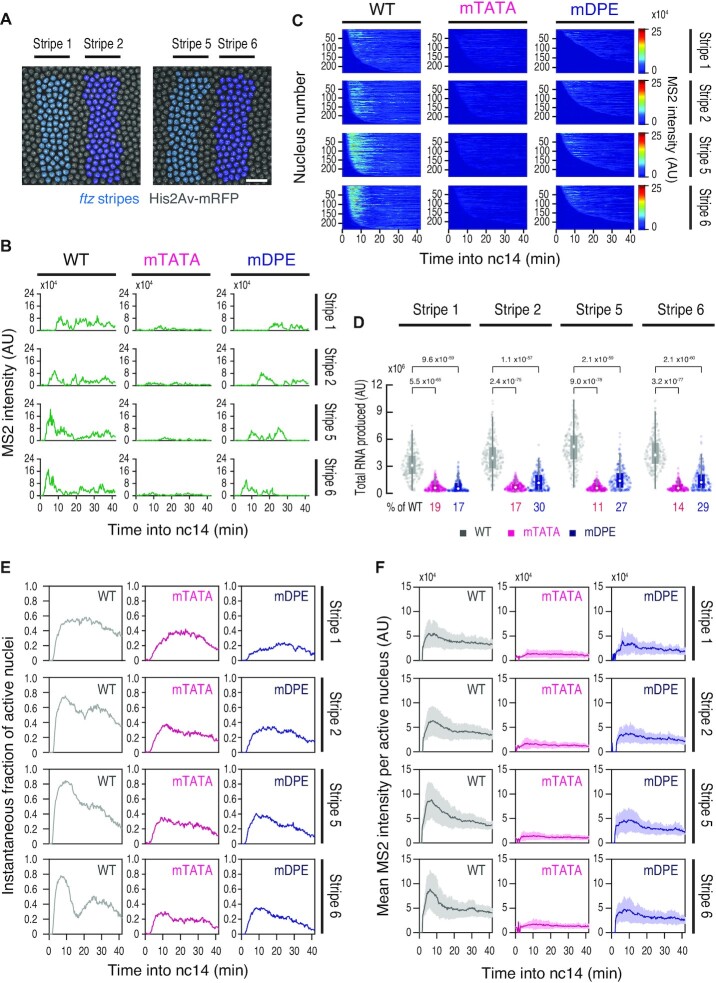
TATA and DPE differentially regulate *ftz* transcription. (**A**, left) False-coloring of nuclei at stripe 1 (cyan) and stripe 2 (blue). (A, right) False-coloring of nuclei at stripe 5 (cyan) and stripe 6 (blue). The maximum projected image of a histone marker (His2Av-mRFP) is shown in gray. Images are oriented with anterior to the left. Scale bar indicates 20 μm. (**B**) Representative trajectories of transcription activity of *ftz-MS2* complementation alleles in individual nuclei. AU; arbitrary unit. (**C**) MS2 trajectories for all analyzed nuclei. Each row represents the MS2 trajectory for a single nucleus. A total of 234, 236 and 227 nuclei at stripe 1, 248, 241 and 242 nuclei at stripe 2, 243, 238 and 240 nuclei at stripe 5, and 232, 239 and 240 nuclei at stripe 6 were analyzed from three independent embryos for the *ftz-MS2* with WT, mTATA and mDPE core promoter, respectively. Nuclei were ordered by their onset of transcription in nc14. AU; arbitrary unit. (**D**) Boxplots showing the distribution of total output. The box indicates the lower (25%) and upper (75%) quantile and the open circle indicates the median. Whiskers extend to the most extreme, non-outlier data points. A total of 234, 236 and 227 nuclei at stripe 1, 248, 241 and 242 nuclei at stripe 2, 243, 238 and 240 nuclei at stripe 5, and 232, 239 and 240 nuclei at stripe 6 were analyzed from three independent embryos for the *ftz-MS2* with WT, mTATA and mDPE core promoter, respectively. Median values relative to the *ftz-MS2* with WT core promoter are shown at the bottom. The *P*-values of two-sided Wilcoxon rank sum test are shown at the top. AU; arbitrary unit. (**E**) Instantaneous fraction of actively transcribing nuclei at each expression domain. A total of 234, 236 and 227 nuclei at stripe 1, 248, 241 and 242 nuclei at stripe 2, 243, 238 and 240 nuclei at stripe 5, and 232, 239 and 240 nuclei at stripe 6 were analyzed from three independent embryos for the *ftz-MS2* with WT, mTATA and mDPE core promoter, respectively. (**F**) Mean MS2 intensity per actively transcribing nucleus at each expression domain. A total of 234, 236 and 227 nuclei at stripe 1, 248, 241 and 242 nuclei at stripe 2, 243, 238 and 240 nuclei at stripe 5, and 232, 239 and 240 nuclei at stripe 6 were analyzed from three independent embryos for the *ftz-MS2* with WT, mTATA and mDPE core promoter, respectively. Shades represent the standard deviation of the mean across active nuclei at a given time.

To elucidate the molecular basis underlying these phenotypes, we carried out live-imaging analysis of individual *ftz-MS2* complementation alleles. We focused on anterior stripe 1/2 and posterior stripe 5/6 (Figure 7A, Supplementary Movie S6) because they are known to be regulated by different enhancers located 5′ and 3′ of the gene (Supplementary Figure S12A) (64,68). While both the mTATA and mDPE *ftz* alleles failed to restore *en* expression and the formation of body segments (Figure 6D and F), their MS2 profiles differ dramatically. The mDPE allele produced delayed discontinuous but strong bursting activity (Figure 7B and C), whereas the mTATA allele further led to an overall diminishment of MS2 intensity in all analyzed stripes (Figure 7B and C). As a consequence, the mTATA allele exhibited a ∼81–89% reduction in the total RNA production, while the mDPE allele showed a more modest reduction (Figure 7D). We then characterized the functional parameters of MS2 trajectories. As we previously reported (25), individual bursting events were hard to be discerned due to the continuity of bursting activities (Figure 7B), suggesting that the endogenous genomic configurations are highly optimized for efficient production of transcriptional bursting within a short period of time. To minimize ambiguity in defining individual bursting events, we quantified the instantaneous fraction of active nuclei (Figure 7E) and the mean MS2 intensity in actively transcribing nuclei (Figure 7F). We found that mutation of TATA and DPE both diminish the fraction of active nuclei (Figure 7E) and also delay the onset of transcription (Supplementary Figure S12B and C). Importantly, the level of MS2 intensity was more severely reduced in the mTATA allele (Figure 7F), suggesting that the TATA mutation reduces the number of Pol II entering into productive elongation even when core promoter in an active state. These profiles were similar to those of mDPE and mTATA DSCP linked to the *sna* shadow enhancer at the synthetic locus (Supplementary Figure S12D and E). We therefore suggest that DPE and TATA differentially regulate the enhancer responsiveness by changing the efficiency and the strength of burst induction. Lastly, to determine how misregulation of bursting activities affects the stripe formation, we computationally reconstituted the spatial patterning of *ftz* by calculating the total RNA production at each nucleus and the *ftz* mRNA turnover rate in early embryos (half-life: 7 min) (32). Although the mDPE allele exhibited somewhat stronger MS2 activities than the mTATA (Figure 7B–F), both resulted in highly sporadic stripe patterns (Supplementary Figure S13). Thus, we concluded that the endogenous configuration of *ftz* core promoter is essential for the uniform expression at the stripe regions to ensure proper control of downstream genes such as *en* during embryo segmentation.

## DISCUSSION

### Molecular mechanism of burst regulation by core promoters

In this study, we provided evidence that each core promoter element differentially modulates the responsiveness to enhancers in early *Drosophila* embryos. Our data suggest that Inr, MTE and DPE mainly affect the frequency of transcriptional bursting by changing the timing of first burst and the continuity of subsequent bursts (Figure 1). On the other hand, TATA mutation dramatically diminished overall transcription activity including the bursting amplitude (Figures 2 and 3). Our genome engineering approach further revealed that the endogenous *ftz* core promoter requires both TATA and DPE to initiate rapid and productive transcription upon activation by stripe enhancers (Figures 6 and 7). We also show that each element can exert its function independently of enhancer-promoter distances or differential combinations of enhancer-promoter pairs (Supplementary Figures S5, S6, S8 and S10). Importantly, recent cryo-EM studies have revealed that TFIID recognizes the core promoter in a stepwise fashion (11,12,70), with initial contact with Inr, MTE and DPE through TAF1/7 and TAF2 subunits, followed by dynamic structural rearrangement that facilitates recognition of the upstream TATA by TBP. We speculate that the rate of initial TFIID loading onto the downstream core promoter region mainly affects the timing and the frequency, and the subsequent TBP loading onto the upstream TATA helps to further increase the bursting amplitude. Supporting this view, a recent biochemical study suggested that downstream core promoter interactions of TFIID increase the efficiency of transcription reinitiation in yeast (71). It was also reported that TATA and Inr can differentially modulate the burst size and the frequency during PD1 expression in primary B-cells (72), although this study focused on their function as a negative regulator of transcription. More recently, it was reported that the loss of TATA or downstream elements alters entire conformation of TFIID by changing molecular spacing between TBP and TAFs (73,74). They further showed that core promoters undergo two distinct PIC assembly pathways depending on the initial conformation of the promoter-bound TFIID. Thus, dynamic structural rearrangements of TFIID during the assembly of PIC can also contribute to differential bursting profiles seen in this study. As a limitation of current study, it is important to note that we engineered core promoter elements by introducing mutations that alternate entire sequences within individual motifs. Future functional studies are needed to fully elucidate the roles of core promoters by analyzing intermediates between the most optimized and dysregulated motifs in the context of various core promoter sequences. We believe that our study will serve as a critical starting point toward understanding of how temporal dynamics of gene expression is encoded in the eukaryotic genome.

### The role of promoter-proximal Zelda sites in burst regulation

During early development of *Drosophila* embryos, a zinc-finger DNA binding protein Zelda plays an essential role in zygotic genome activation (75). At the enhancer regions, it is well established that Zelda exerts its pioneering activity by lowering nucleosome barriers to assist subsequent recruitment of transcription factors and coactivators (53,55). Importantly, our data showed that the addition of Zelda sites at the core promoter region augments both the amplitude and the frequency of transcriptional bursting (Figures 4 and 5). In analogy to Zelda's function at the enhancer regions, it is conceivable that the promoter-opening by Zelda contributes to the initial TFIID recruitment and the subsequent TBP loading at the core promoter region. It has been previously shown that Zelda is pre-loaded onto a thousand of promoter regions prior to zygotic genome activation in early *Drosophila* embryos (15). Thus, it appears that promoter-proximal Zelda sites increase the responsiveness to enhancers by making core promoters poised for activation even before distal enhancers start to drive transcription. We suggest that multi-layered mechanisms of Zelda and other elements help to diversify the enhancer responsiveness of core promoters across the genome because there are substantial variations in the composition of core promoter elements among Pol II-transcribed genes. For example, it is estimated that only a small fraction of protein coding genes contain TATA, both in human and *Drosophila* (17–19). Importantly, our data showed that the TATA-less core promoter of the Hox gene *lab* is naturally depleted of TATA to limit the level of total RNA production by reducing the amplitude of transcriptional bursting (Figure 3). As suboptimal transcription factor binding sites are important for tissue-specific gene activation by enhancers (76,77), suboptimal core promoter architectures might play a key role in ensuring the spatial and temporal specificity of gene expression during animal development. Alternatively, it can be possible that a subset of enhancers is capable of driving strong bursts even when TATA or other elements are not present as suggested by previous enhancer trapping assay in *Drosophila* (21). In this regard, our data suggests that the promoter-proximal Zelda sites can help to compensate weak activities of TATA-less core promoters in the *Drosophila* genome (Figure 5).

### *Drosophila* TATA regulates minute-scale bursting activities

Regulation of transcriptional bursting by core promoter elements seems to be a common mechanism conserved across species. Analysis of actin family genes in *Dictyostelium* cells also reported that swapping of entire promoter sequence can alter profiles of transcriptional bursting (78). Previous studies showed that TATA mutation reduces the burst size and gene expression noise in yeast and mammals (3,42–48). Consistently, our data also showed that TATA largely affects the amplitude of transcriptional bursting (Figures 2 and 3). However, there seems to be a clear difference in a time-scale of bursting activities that TATA impacts between species. In mouse and human cells, transcriptional bursts are often separated by refractory period of several hours (e.g. 79–82). Importantly, live-imaging analysis of HIV-1 reporter gene suggested that the mutation of TATA specifically affects hour-scale, but not minute-scale, transcriptional bursting in HeLa cells (47). Recent single-cell RNA-seq study also suggested that TATA affects hour-scale bursting activities in mouse and human cultured cells (3). In contrast, TATA clearly influenced minute-scale bursting profiles in our *Drosophila* system (Figures 2 and 3). Thus, it appears that *Drosophila* embryos use TATA in a different way from mammalian systems to regulate bursting activities at a finer time-scale, which may help to control dynamic ON/OFF patterns of gene expression during rapid processes of early embryogenesis.

## DATA AVAILABILITY

Original live imaging data have been deposited to Zenodo database (https://doi.org/10.5281/zenodo.5025660).

## Supplementary Material

gkab1177_Supplemental_Files

## References

[1] Bartman C.R. , Hsu S.C., Hsiung C.C., Raj A., Blobel G.A. Enhancer regulation of transcriptional bursting parameters revealed by forced chromatin looping. Mol. Cell. 2016; 62:237–247. 27067601 10.1016/j.molcel.2016.03.007 PMC4842148

[2] Fukaya T. , Lim B., Levine M. Enhancer control of transcriptional bursting. Cell. 2016; 166:358–368. 27293191 10.1016/j.cell.2016.05.025 PMC4970759

[3] Larsson A.J.M. , Johnsson P., Hagemann-Jensen M., Hartmanis L., Faridani O.R., Reinius B., Segerstolpe A., Rivera C.M., Ren B., Sandberg R. Genomic encoding of transcriptional burst kinetics. Nature. 2019; 565:251–254. 30602787 10.1038/s41586-018-0836-1 PMC7610481

[4] Vo Ngoc L. , Kassavetis G.A., Kadonaga J.T. The RNA polymerase II core promoter in *Drosophila*. Genetics. 2019; 212:13–24. 31053615 10.1534/genetics.119.302021 PMC6499525

[5] Goldberg M.L. Sequence Analysis of *Drosophila* Histone Genes. 1979; Stanford, CA Stanford University Ph.D. Dissertation.

[6] Burke T.W. , Kadonaga J.T. Drosophila TFIID binds to a conserved downstream basal promoter element that is present in many TATA-box-deficient promoters. Genes Dev. 1996; 10:711–724. 8598298 10.1101/gad.10.6.711

[7] Lim C.Y. , Santoso B., Boulay T., Dong E., Ohler U., Kadonaga J.T. The MTE, a new core promoter element for transcription by RNA polymerase II. Genes Dev. 2004; 18:1606–1617. 15231738 10.1101/gad.1193404 PMC443522

[8] Smale S.T. , Baltimore D The “initiator” as a transcription control element. Cell. 1989; 57:103–113. 2467742 10.1016/0092-8674(89)90176-1

[9] Burke T.W. , Kadonaga J.T. The downstream core promoter element, DPE, is conserved from *Drosophila* to humans and is recognized by TAFII60 of *Drosophila*. Genes Dev. 1997; 11:3020–3031. 9367984 10.1101/gad.11.22.3020 PMC316699

[10] Chalkley G.E. , Verrijzer C.P. DNA binding site selection by RNA polymerase II TAFs: a TAF(II)250-TAF(II)150 complex recognizes the initiator. EMBO J. 1999; 18:4835–4845. 10469661 10.1093/emboj/18.17.4835 PMC1171555

[11] Louder R.K. , He Y., Lopez-Blanco J.R., Fang J., Chacon P., Nogales E. Structure of promoter-bound TFIID and model of human pre-initiation complex assembly. Nature. 2016; 531:604–609. 27007846 10.1038/nature17394 PMC4856295

[12] Patel A.B. , Louder R.K., Greber B.J., Grunberg S., Luo J., Fang J., Liu Y., Ranish J., Hahn S., Nogales E. Structure of human TFIID and mechanism of TBP loading onto promoter DNA. Science. 2018; 362:eaau8872. 30442764 10.1126/science.aau8872 PMC6446905

[13] Theisen J.W. , Lim C.Y., Kadonaga J.T. Three key subregions contribute to the function of the downstream RNA polymerase II core promoter. Mol. Cell. Biol. 2010; 30:3471–3479. 20457814 10.1128/MCB.00053-10 PMC2897566

[14] Chen K. , Johnston J., Shao W., Meier S., Staber C., Zeitlinger J. A global change in RNA polymerase II pausing during the *Drosophila* midblastula transition. Elife. 2013; 2:e00861. 23951546 10.7554/eLife.00861 PMC3743134

[15] Harrison M.M. , Li X.Y., Kaplan T., Botchan M.R., Eisen M.B. Zelda binding in the early *Drosophila melanogaster* embryo marks regions subsequently activated at the maternal-to-zygotic transition. PLoS Genet. 2011; 7:e1002266. 22028662 10.1371/journal.pgen.1002266 PMC3197655

[16] Hendrix D.A. , Hong J.W., Zeitlinger J., Rokhsar D.S., Levine M.S. Promoter elements associated with RNA Pol II stalling in the *Drosophila* embryo. Proc. Natl. Acad. Sci. U.S.A. 2008; 105:7762–7767. 18505835 10.1073/pnas.0802406105 PMC2396556

[17] Chen Z.X. , Sturgill D., Qu J., Jiang H., Park S., Boley N., Suzuki A.M., Fletcher A.R., Plachetzki D.C., FitzGerald P.C. et al. Comparative validation of the *D. melanogaster* modENCODE transcriptome annotation. Genome Res. 2014; 24:1209–1223. 24985915 10.1101/gr.159384.113 PMC4079975

[18] FitzGerald P.C. , Sturgill D., Shyakhtenko A., Oliver B., Vinson C. Comparative genomics of *Drosophila* and human core promoters. Genome Biol. 2006; 7:R53. 16827941 10.1186/gb-2006-7-7-r53 PMC1779564

[19] Ohler U. , Liao G.C., Niemann H., Rubin G.M. Computational analysis of core promoters in the *Drosophila* genome. Genome Biol. 2002; 3:RESEARCH0087. 12537576 10.1186/gb-2002-3-12-research0087 PMC151189

[20] Arnold C.D. , Zabidi M.A., Pagani M., Rath M., Schernhuber K., Kazmar T., Stark A. Genome-wide assessment of sequence-intrinsic enhancer responsiveness at single-base-pair resolution. Nat. Biotechnol. 2017; 35:136–144. 28024147 10.1038/nbt.3739 PMC5870828

[21] Butler J.E. , Kadonaga J.T. Enhancer-promoter specificity mediated by DPE or TATA core promoter motifs. Genes Dev. 2001; 15:2515–2519. 11581157 10.1101/gad.924301 PMC312797

[22] Ohtsuki S. , Levine M., Cai H.N. Different core promoters possess distinct regulatory activities in the *Drosophila* embryo. Genes Dev. 1998; 12:547–556. 9472023 10.1101/gad.12.4.547 PMC316525

[23] Zabidi M.A. , Arnold C.D., Schernhuber K., Pagani M., Rath M., Frank O., Stark A. Enhancer-core-promoter specificity separates developmental and housekeeping gene regulation. Nature. 2015; 518:556–559. 25517091 10.1038/nature13994 PMC6795551

[24] Furlong E.E.M. , Levine M. Developmental enhancers and chromosome topology. Science. 2018; 361:1341–1345. 30262496 10.1126/science.aau0320 PMC6986801

[25] Yokoshi M. , Segawa K., Fukaya T. Visualizing the role of boundary elements en Enhancer-promoter communication. Mol. Cell. 2020; 78:224–235. 32109364 10.1016/j.molcel.2020.02.007

[26] Venken K.J. , He Y., Hoskins R.A., Bellen H.J. P[acman]: a BAC transgenic platform for targeted insertion of large DNA fragments in *D. melanogaster*. Science. 2006; 314:1747–1751. 17138868 10.1126/science.1134426

[27] Bischof J. , Maeda R.K., Hediger M., Karch F., Basler K. An optimized transgenesis system for *Drosophila* using germ-line-specific phiC31 integrases. Proc. Natl. Acad. Sci. U.S.A. 2007; 104:3312–3317. 17360644 10.1073/pnas.0611511104 PMC1805588

[28] Ringrose L. Transgenesis in *Drosophila* melanogaster. Methods Mol. Biol. 2009; 561:3–19. 19504061 10.1007/978-1-60327-019-9_1

[29] Ren X.J. , Sun J., Housden B.E., Hu Y.H., Roesel C., Lin S.L., Liu L.P., Yang Z.H., Mao D.C., Sun L.Z. et al. Optimized gene editing technology for *Drosophila melanogaster* using germ line-specific Cas9. Proc. Natl. Acad. Sci. U.S.A. 2013; 110:19012–19017. 24191015 10.1073/pnas.1318481110 PMC3839733

[30] Dufourt J. , Trullo A., Hunter J., Fernandez C., Lazaro J., Dejean M., Morales L., Nait-Amer S., Schulz K.N., Harrison M.M. et al. Temporal control of gene expression by the pioneer factor Zelda through transient interactions in hubs. Nat. Commun. 2018; 9:5194. 30518940 10.1038/s41467-018-07613-z PMC6281682

[31] Martin R.M. , Rino J., Carvalho C., Kirchhausen T., Carmo-Fonseca M. Live-cell visualization of pre-mRNA splicing with single-molecule sensitivity. Cell Rep. 2013; 4:1144–1155. 24035393 10.1016/j.celrep.2013.08.013 PMC3805459

[32] Edgar B.A. , Weir M.P., Schubiger G., Kornberg T. Repression and turnover pattern *fushi tarazu* RNA in the early *Drosophila* embryo. Cell. 1986; 47:747–754. 3096577 10.1016/0092-8674(86)90517-9

[33] Garcia H.G. , Tikhonov M., Lin A., Gregor T. Quantitative imaging of transcription in living *Drosophila* embryos links polymerase activity to patterning. Curr. Biol. 2013; 23:2140–2145. 24139738 10.1016/j.cub.2013.08.054 PMC3828032

[34] Lucas T. , Ferraro T., Roelens B., De Las Heras Chanes J., Walczak A.M., Coppey M., Dostatni N Live imaging of bicoid-dependent transcription in *Drosophila* embryos. Curr. Biol. 2013; 23:2135–2139. 24139736 10.1016/j.cub.2013.08.053

[35] Pfeiffer B.D. , Jenett A., Hammonds A.S., Ngo T.T., Misra S., Murphy C., Scully A., Carlson J.W., Wan K.H., Laverty T.R. et al. Tools for neuroanatomy and neurogenetics in *Drosophila*. Proc. Natl. Acad. Sci. U.S.A. 2008; 105:9715–9720. 18621688 10.1073/pnas.0803697105 PMC2447866

[36] Dunipace L. , Ozdemir A., Stathopoulos A. Complex interactions between cis-regulatory modules in native conformation are critical for *Drosophila snail* expression. Development. 2011; 138:4075–4084. 21813571 10.1242/dev.069146 PMC3160101

[37] Perry M.W. , Boettiger A.N., Bothma J.P., Levine M. Shadow enhancers foster robustness of *Drosophila* gastrulation. Curr. Biol. 2010; 20:1562–1567. 20797865 10.1016/j.cub.2010.07.043 PMC4257487

[38] Groth A.C. , Fish M., Nusse R., Calos M.P. Construction of transgenic *Drosophila* by using the site-specific integrase from phage phiC31. Genetics. 2004; 166:1775–1782. 15126397 10.1534/genetics.166.4.1775 PMC1470814

[39] Kutach A.K. , Kadonaga J.T. The downstream promoter element DPE appears to be as widely used as the TATA box in *Drosophila* core promoters. Mol. Cell. Biol. 2000; 20:4754–4764. 10848601 10.1128/mcb.20.13.4754-4764.2000 PMC85905

[40] Cai H. , Levine M. Modulation of enhancer-promoter interactions by insulators in the *Drosophila* embryo. Nature. 1995; 376:533–536. 7637789 10.1038/376533a0

[41] Ip Y.T. , Park R.E., Kosman D., Bier E., Levine M. The dorsal gradient morphogen regulates stripes of *rhomboid* expression in the presumptive neuroectoderm of the *Drosophila* embryo. Genes Dev. 1992; 6:1728–1739. 1325394 10.1101/gad.6.9.1728

[42] Ochiai H. , Hayashi T., Umeda M., Yoshimura M., Harada A., Shimizu Y., Nakano K., Saitoh N., Liu Z., Yamamoto T. et al. Genome-wide kinetic properties of transcriptional bursting in mouse embryonic stem cells. Sci. Adv. 2020; 6:eaaz6699. 32596448 10.1126/sciadv.aaz6699 PMC7299619

[43] Blake W.J. , Balazsi G., Kohanski M.A., Isaacs F.J., Murphy K.F., Kuang Y., Cantor C.R., Walt D.R., Collins J.J. Phenotypic consequences of promoter-mediated transcriptional noise. Mol. Cell. 2006; 24:853–865. 17189188 10.1016/j.molcel.2006.11.003

[44] Hornung G. , Bar-Ziv R., Rosin D., Tokuriki N., Tawfik D.S., Oren M., Barkai N. Noise-mean relationship in mutated promoters. Genome Res. 2012; 22:2409–2417. 22820945 10.1101/gr.139378.112 PMC3514670

[45] Murphy K.F. , Adams R.M., Wang X., Balazsi G., Collins J.J. Tuning and controlling gene expression noise in synthetic gene networks. Nucleic Acids Res. 2010; 38:2712–2726. 20211838 10.1093/nar/gkq091 PMC2860118

[46] Patwardhan R.P. , Lee C., Litvin O., Young D.L., Pe’er D., Shendure J. High-resolution analysis of DNA regulatory elements by synthetic saturation mutagenesis. Nat. Biotechnol. 2009; 27:1173–1175. 19915551 10.1038/nbt.1589 PMC2849652

[47] Tantale K. , Mueller F., Kozulic-Pirher A., Lesne A., Victor J.M., Robert M.C., Capozi S., Chouaib R., Backer V., Mateos-Langerak J. et al. A single-molecule view of transcription reveals convoys of RNA polymerases and multi-scale bursting. Nat. Commun. 2016; 7:12248. 27461529 10.1038/ncomms12248 PMC4974459

[48] Raser J.M. , O'Shea E.K Control of stochasticity in eukaryotic gene expression. Science. 2004; 304:1811–1814. 15166317 10.1126/science.1098641 PMC1410811

[49] Juven-Gershon T. , Hsu J.Y., Kadonaga J.T. Caudal, a key developmental regulator, is a DPE-specific transcriptional factor. Genes Dev. 2008; 22:2823–2830. 18923080 10.1101/gad.1698108 PMC2569877

[50] Busturia A. , Bienz M. Silencers in abdominal-B, a homeotic *Drosophila* gene. EMBO J. 1993; 12:1415–1425. 8096812 10.1002/j.1460-2075.1993.tb05785.x PMC413353

[51] Lee C. , Li X., Hechmer A., Eisen M., Biggin M.D., Venters B.J., Jiang C., Li J., Pugh B.F., Gilmour D.S. NELF and GAGA factor are linked to promoter-proximal pausing at many genes in *Drosophila*. Mol. Cell. Biol. 2008; 28:3290–3300. 18332113 10.1128/MCB.02224-07 PMC2423147

[52] Fuda N.J. , Guertin M.J., Sharma S., Danko C.G., Martins A.L., Siepel A., Lis J.T. GAGA factor maintains nucleosome-free regions and has a role in RNA polymerase II recruitment to promoters. PLoS Genet. 2015; 11:e1005108. 25815464 10.1371/journal.pgen.1005108 PMC4376892

[53] Sun Y. , Nien C.Y., Chen K., Liu H.Y., Johnston J., Zeitlinger J., Rushlow C. Zelda overcomes the high intrinsic nucleosome barrier at enhancers during *Drosophila* zygotic genome activation. Genome Res. 2015; 25:1703–1714. 26335633 10.1101/gr.192542.115 PMC4617966

[54] Tsukiyama T. , Wu C. Purification and properties of an ATP-dependent nucleosome remodeling factor. Cell. 1995; 83:1011–1020. 8521501 10.1016/0092-8674(95)90216-3

[55] Foo S.M. , Sun Y., Lim B., Ziukaite R., O’Brien K., Nien C.Y., Kirov N., Shvartsman S.Y., Rushlow C.A. Zelda potentiates morphogen activity by increasing chromatin accessibility. Curr. Biol. 2014; 24:1341–1346. 24909324 10.1016/j.cub.2014.04.032 PMC4075064

[56] Yamada S. , Whitney P.H., Huang S.K., Eck E.C., Garcia H.G., Rushlow C.A. The Drosophila pioneer factor zelda modulates the nuclear microenvironment of a dorsal target enhancer to potentiate transcriptional output. Curr. Biol. 2019; 29:1387–1393. 30982648 10.1016/j.cub.2019.03.019 PMC6702943

[57] Ohtsuki S. , Levine M. GAGA mediates the enhancer blocking activity of the eve promoter in the *Drosophila* embryo. Genes Dev. 1998; 12:3325–3330. 9808619 10.1101/gad.12.21.3325 PMC317233

[58] Ling J. , Umezawa K.Y., Scott T., Small S. Bicoid-dependent activation of the target gene hunchback requires a two-motif sequence code in a specific basal promoter. Mol. Cell. 2019; 75:1178–1187. 31402096 10.1016/j.molcel.2019.06.038 PMC6754290

[59] Kuroiwa A. , Hafen E., Gehring W.J. Cloning and transcriptional analysis of the segmentation gene *fushi tarazu* of *Drosophila*. Cell. 1984; 37:825–831. 6430567 10.1016/0092-8674(84)90417-3

[60] Nüsslein-Volhard C. , Wieschaus E., Jürgens G. Segmentierung bei *Drosophila* — eine genetische Analyse. Verh. Ges. Dtsch. Zool. 1982; 75:91–104.

[61] Wakimoto B.T. , Kaufman T.C. Analysis of larval segmentation in lethal genotypes associated with the antennapedia gene complex in *Drosophila melanogaster*. Dev. Biol. 1981; 81:51–64. 6780397 10.1016/0012-1606(81)90347-x

[62] Wakimoto B.T. , Turner F.R., Kaufman T.C. Defects in embryogenesis in mutants associated with the antennapedia gene complex of *Drosophila melanogaster*. Dev. Biol. 1984; 102:147–172. 6421639 10.1016/0012-1606(84)90182-9

[63] Hafen E. , Kuroiwa A., Gehring W.J. Spatial distribution of transcripts from the segmentation gene *fushi tarazu* during *Drosophila* embryonic development. Cell. 1984; 37:833–841. 6430568 10.1016/0092-8674(84)90418-5

[64] Calhoun V.C. , Levine M. Long-range enhancer-promoter interactions in the *Scr-Antp* interval of the *Drosophila* Antennapedia complex. Proc. Natl. Acad. Sci. U.S.A. 2003; 100:9878–9883. 12909726 10.1073/pnas.1233791100 PMC187872

[65] Hiromi Y. , Gehring W.J. Regulation and function of the *Drosophila* segmentation gene *fushi tarazu*. Cell. 1987; 50:963–974. 2887293 10.1016/0092-8674(87)90523-x

[66] Hiromi Y. , Kuroiwa A., Gehring W.J. Control elements of the *Drosophila* segmentation gene *fushi tarazu*. Cell. 1985; 43:603–613. 3935327 10.1016/0092-8674(85)90232-6

[67] Pick L. , Schier A., Affolter M., Schmidt-Glenewinkel T., Gehring W.J. Analysis of the *ftz* upstream element: germ layer-specific enhancers are independently autoregulated. Genes Dev. 1990; 4:1224–1239. 1976571 10.1101/gad.4.7.1224

[68] Schroeder M.D. , Greer C., Gaul U. How to make stripes: deciphering the transition from non-periodic to periodic patterns in *Drosophila* segmentation. Development. 2011; 138:3067–3078. 21693522 10.1242/dev.062141 PMC3119311

[69] Macdonald P.M. , Struhl G. A molecular gradient in early *Drosophila* embryos and its role in specifying the body pattern. Nature. 1986; 324:537–545. 2878369 10.1038/324537a0

[70] Cianfrocco M.A. , Kassavetis G.A., Grob P., Fang J., Juven-Gershon T., Kadonaga J.T., Nogales E. Human TFIID binds to core promoter DNA in a reorganized structural state. Cell. 2013; 152:120–131. 23332750 10.1016/j.cell.2012.12.005 PMC3552382

[71] Joo Y.J. , Ficarro S.B., Soares L.M., Chun Y., Marto J.A., Buratowski S. Downstream promoter interactions of TFIID TAFs facilitate transcription reinitiation. Genes Dev. 2017; 31:2162–2174. 29203645 10.1101/gad.306324.117 PMC5749164

[72] Hendy O. , Campbell J. Jr., Weissman J.D., Larson D.R., Singer D.S Differential context-specific impact of individual core promoter elements on transcriptional dynamics. Mol. Biol. Cell. 2017; 28:3360–3370. 28931597 10.1091/mbc.E17-06-0408 PMC5687036

[73] Chen X. , Qi Y., Wu Z., Wang X., Li J., Zhao D., Hou H., Li Y., Yu Z., Liu W. et al. Structural insights into preinitiation complex assembly on core promoters. Science. 2021; 372:eaba8490. 33795473 10.1126/science.aba8490

[74] Chen X. , Yin X., Li J., Wu Z., Qi Y., Wang X., Liu W., Xu Y. Structures of the human Mediator and Mediator-bound preinitiation complex. Science. 2021; 372:eabg0635. 33958484 10.1126/science.abg0635

[75] Liang H.L. , Nien C.Y., Liu H.Y., Metzstein M.M., Kirov N., Rushlow C. The zinc-finger protein Zelda is a key activator of the early zygotic genome in *Drosophila*. Nature. 2008; 456:400–403. 18931655 10.1038/nature07388 PMC2597674

[76] Crocker J. , Abe N., Rinaldi L., McGregor A.P., Frankel N., Wang S., Alsawadi A., Valenti P., Plaza S., Payre F. et al. Low affinity binding site clusters confer hox specificity and regulatory robustness. Cell. 2015; 160:191–203. 25557079 10.1016/j.cell.2014.11.041 PMC4449256

[77] Farley E.K. , Olson K.M., Zhang W., Brandt A.J., Rokhsar D.S., Levine M.S. Suboptimization of developmental enhancers. Science. 2015; 350:325–328. 26472909 10.1126/science.aac6948 PMC4970741

[78] Tunnacliffe E. , Corrigan A.M., Chubb J.R. Promoter-mediated diversification of transcriptional bursting dynamics following gene duplication. Proc. Natl. Acad. Sci. U.S.A. 2018; 115:8364–8369. 30061408 10.1073/pnas.1800943115 PMC6099902

[79] Molina N. , Suter D.M., Cannavo R., Zoller B., Gotic I., Naef F. Stimulus-induced modulation of transcriptional bursting in a single mammalian gene. Proc. Natl. Acad. Sci. U.S.A. 2013; 110:20563–20568. 24297917 10.1073/pnas.1312310110 PMC3870742

[80] Nicolas D. , Zoller B., Suter D.M., Naef F. Modulation of transcriptional burst frequency by histone acetylation. Proc. Natl. Acad. Sci. U.S.A. 2018; 115:7153–7158. 29915087 10.1073/pnas.1722330115 PMC6142243

[81] Rodriguez J. , Ren G., Day C.R., Zhao K., Chow C.C., Larson D.R. Intrinsic dynamics of a human gene reveal the basis of expression heterogeneity. Cell. 2019; 176:213–226. 30554876 10.1016/j.cell.2018.11.026 PMC6331006

[82] Suter D.M. , Molina N., Gatfield D., Schneider K., Schibler U., Naef F. Mammalian genes are transcribed with widely different bursting kinetics. Science. 2011; 332:472–474. 21415320 10.1126/science.1198817

