# Supplementary material for: Dynamic modulation of enhancer responsiveness by core promoter elements in living Drosophila embryos
Source: Nucleic Acids Res. 2021 Dec 13;50(1):92–107. doi: 10.1093/nar/gkab1177 (PMC8754644; doi:10.1093/nar/gkab1177)
Supplement: gkab1177_Supplemental_Files [file gkab1177_supplemental_files.zip › Supplemental_Figure_1108.pdf]

Figure S1

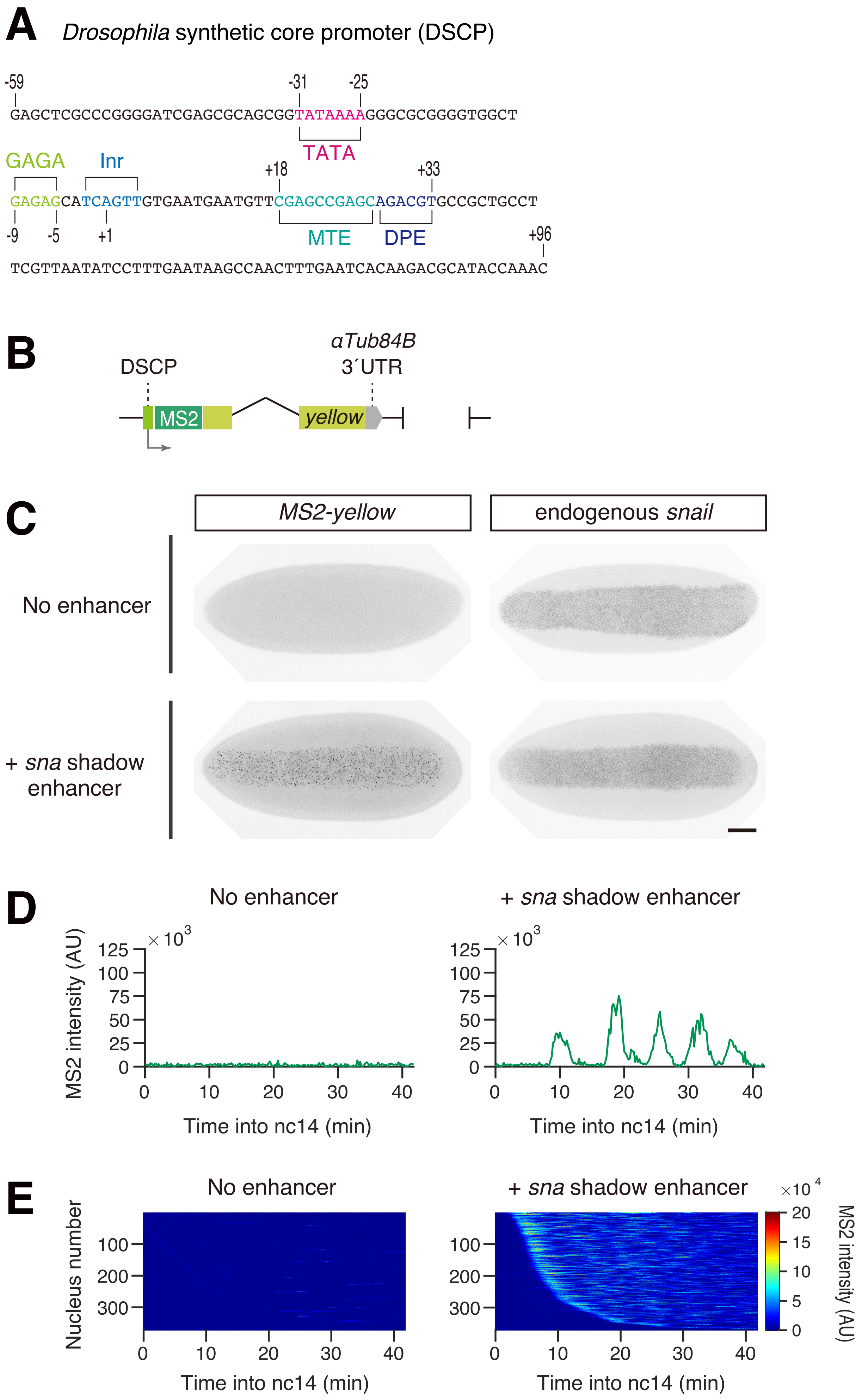
A
Drosophila synthetic core promoter (DSCP)
-59
-31
-25
GAGCTCGCCCGGGGATCGAGCGCAGCGGTATAAAAGGGCGCGGGGTGGCT
TATA
GAGA
Inr
+18
+33
GAGAGCATCAGTTGTGAATGAATGTTCGAGCCGAGCAGACGTGCCGCTGCCT
-9
-5
+1
MTE
DPE
+96
TCGTTAATATCCTTTGAATAAGCCAACTTTGAATCACAAGACGCATACCAAAC
B
αTub84B
3´UTR
DSCP
MS2
yellow
C
MS2-yellow
endogenous snail
No enhancer
+ sna shadow enhancer
D
No enhancer
+ sna shadow enhancer
MS2 intensity (AU)
×10³
125
100
75
50
25
0
0
10
20
30
40
Time into nc14 (min)
E
No enhancer
+ sna shadow enhancer
Nucleus number
100
200
300
0
10
20
30
40
Time into nc14 (min)
×10⁴
20
15
10
5
0
MS2 intensity (AU)

Figure S2

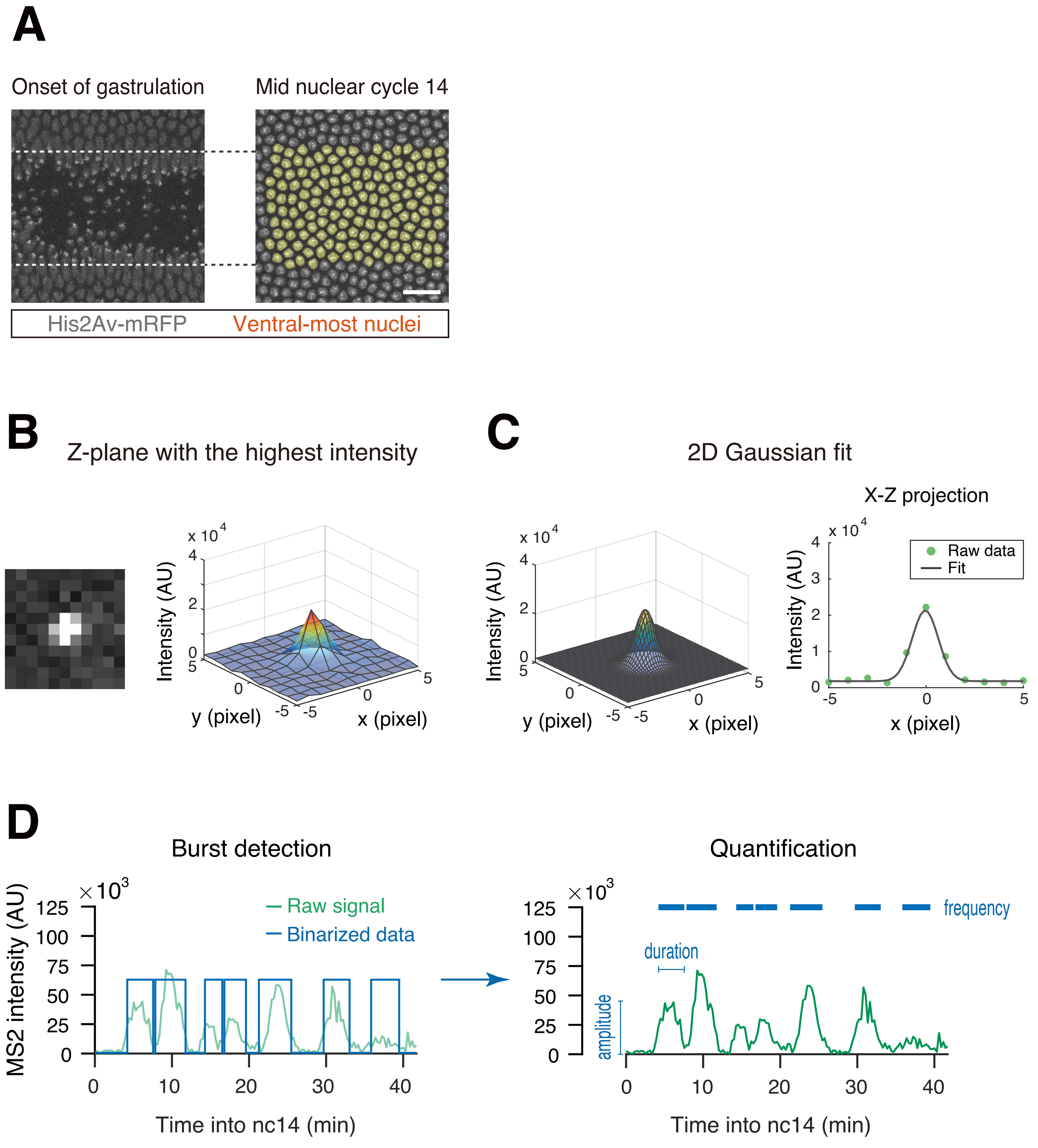
A
Onset of gastrulation
Mid nuclear cycle 14
His2Av-mRFP
Ventral-most nuclei
B
Z-plane with the highest intensity
x 10^4
Intensity (AU)
y (pixel)
x (pixel)
C
2D Gaussian fit
X-Z projection
Raw data
Fit
D
Burst detection
Quantification
MS2 intensity (AU)
×10^3
Raw signal
Binarized data
Time into nc14 (min)
frequency
duration
amplitude

Figure S3

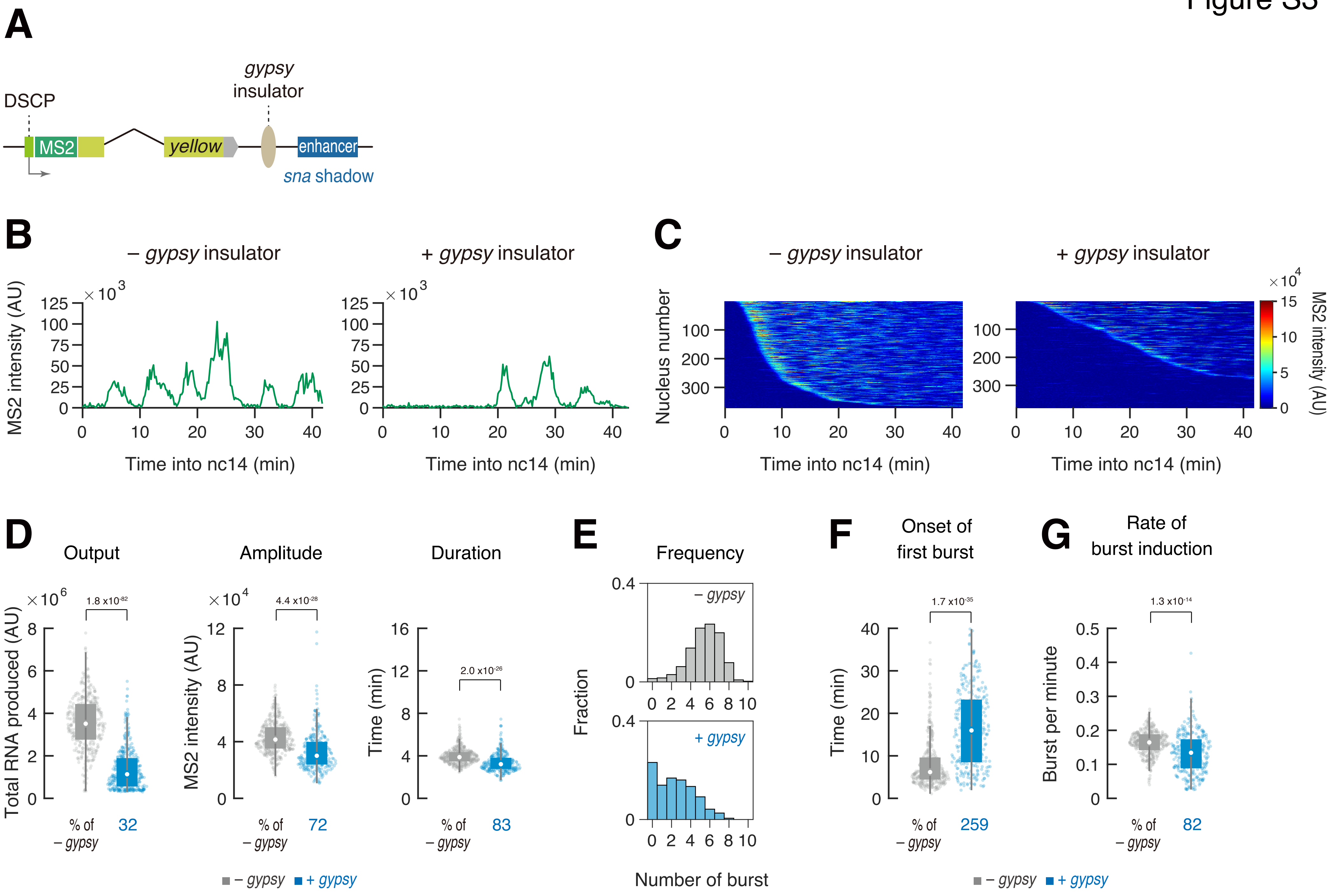

A
DSCP
MS2
yellow
gypsy insulator
enhancer
sna shadow
B
– gypsy insulator
+ gypsy insulator
MS2 intensity (AU)
×10^3
Time into nc14 (min)
C
– gypsy insulator
+ gypsy insulator
Nucleus number
×10^4
MS2 intensity (AU)
Time into nc14 (min)
D
Output
Amplitude
Duration
Total RNA produced (AU)
×10^6
1.8 x10^-82
MS2 intensity (AU)
×10^4
4.4 x10^-28
Time (min)
2.0 x10^-26
% of – gypsy
32
72
83
– gypsy
+ gypsy
E
Frequency
Fraction
– gypsy
+ gypsy
Number of burst
F
Onset of first burst
Time (min)
1.7 x10^-35
259
G
Rate of burst induction
Burst per minute
1.3 x10^-14
82

Figure S4

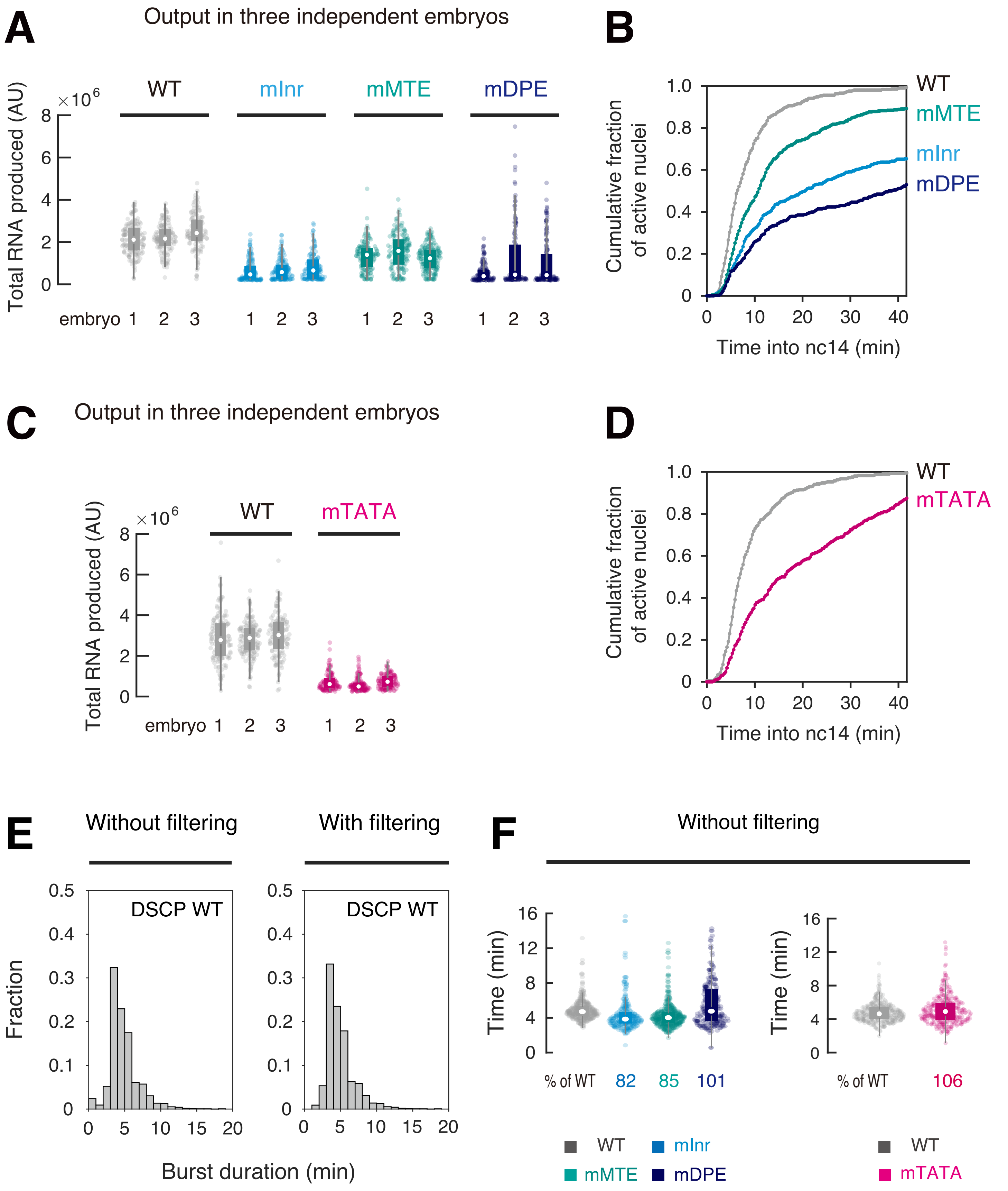
A
Output in three independent embryos
Total RNA produced (AU)
×10^6
WT
mInr
mMTE
mDPE
embryo 1 2 3 1 2 3 1 2 3 1 2 3
B
Cumulative fraction of active nuclei
Time into nc14 (min)
WT
mMTE
mInr
mDPE
C
Output in three independent embryos
Total RNA produced (AU)
×10^6
WT
mTATA
embryo 1 2 3 1 2 3
D
Cumulative fraction of active nuclei
Time into nc14 (min)
WT
mTATA
E
Without filtering
With filtering
Fraction
DSCP WT
DSCP WT
Burst duration (min)
F
Without filtering
Time (min)
% of WT 82 85 101
Time (min)
% of WT 106
WT
mInr
mMTE
mDPE
WT
mTATA

Figure S5

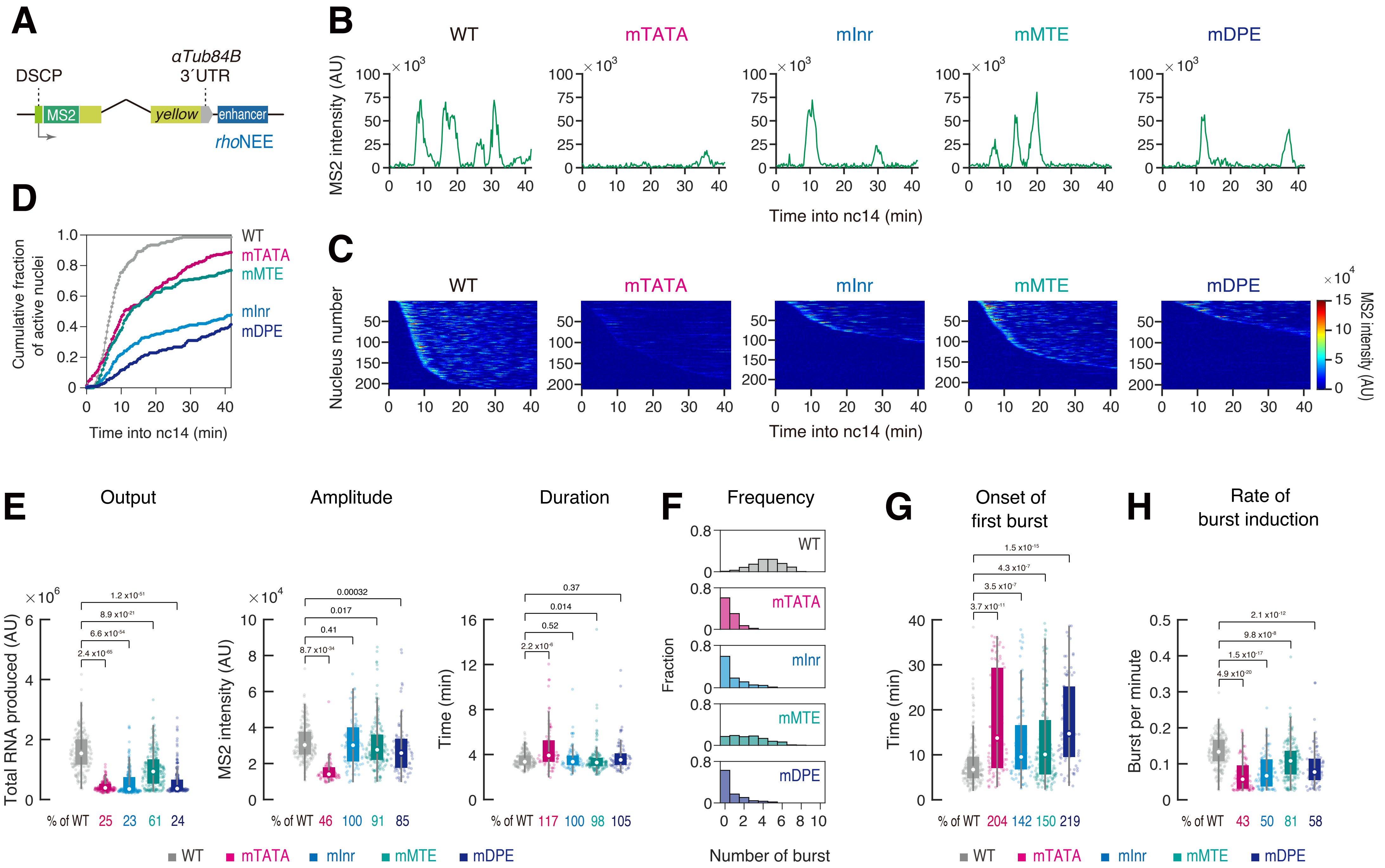
A
DSCP
αTub84B
3´UTR
MS2
yellow
enhancer
rhoNEE
B
WT
mTATA
mInr
mMTE
mDPE
MS2 intensity (AU)
×10^3
Time into nc14 (min)
C
Nucleus number
MS2 intensity (AU)
×10^4
Time into nc14 (min)
D
Cumulative fraction of active nuclei
Time into nc14 (min)
E
Output
Amplitude
Duration
Total RNA produced (AU)
×10^6
MS2 intensity (AU)
×10^4
Time (min)
% of WT 25 23 61 24
% of WT 46 100 91 85
% of WT 117 100 98 105
F
Frequency
Fraction
Number of burst
G
Onset of first burst
Time (min)
% of WT 204 142 150 219
H
Rate of burst induction
Burst per minute
% of WT 43 50 81 58
WT
mTATA
mInr
mMTE
mDPE

Figure S6

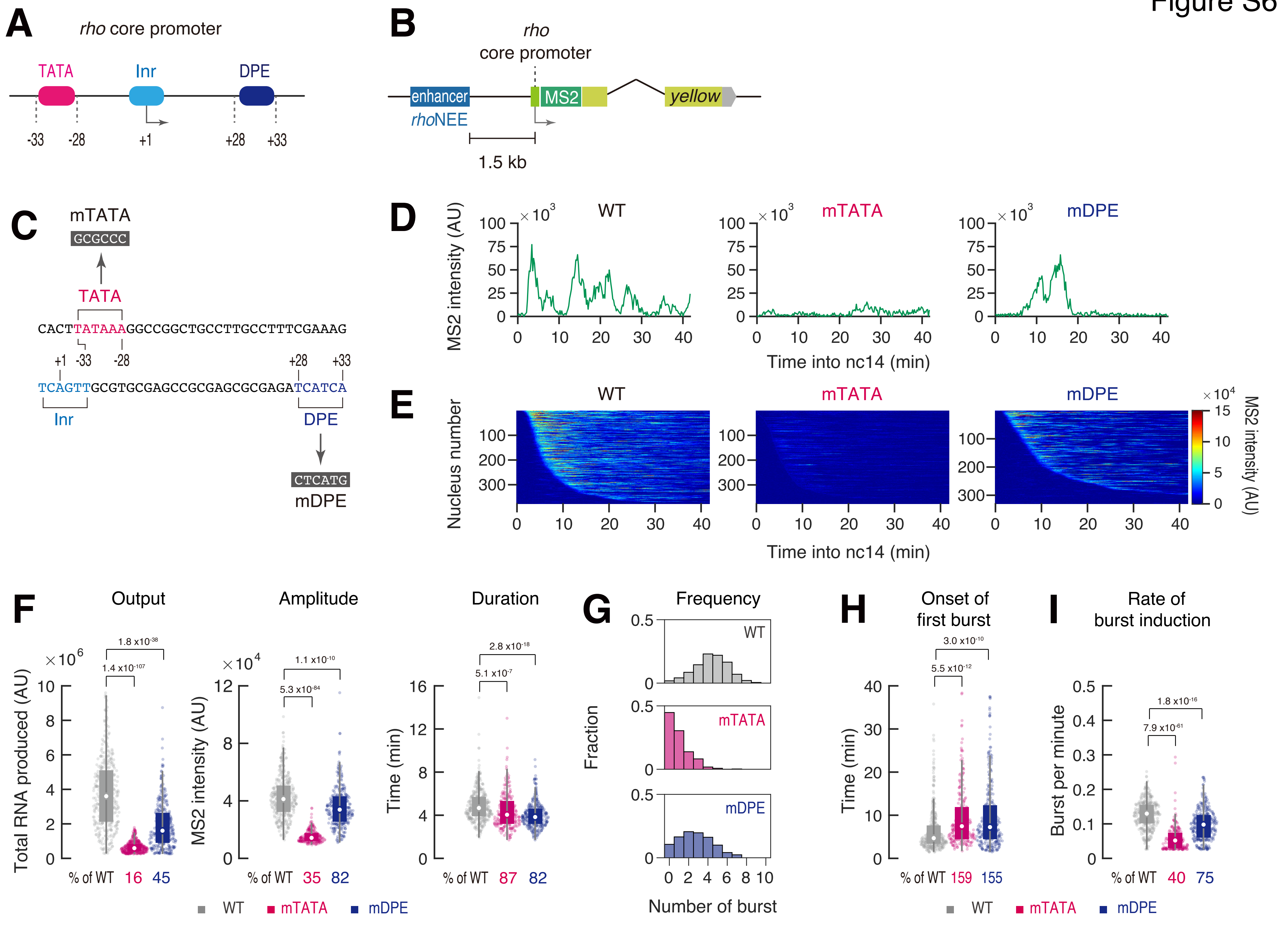

A
rho core promoter
TATA
Inr
DPE
-33
-28
+1
+28
+33
B
rho
core promoter
enhancer
rhoNEE
MS2
yellow
1.5 kb
C
mTATA
GCGCCC
TATA
CACTTATAAAGGCCGGCTGCCTTGCCTTTCGAAAG
+1 -33 -28 +28 +33
TCAGTTGCGTGCGAGCCGCGAGCGCGAGATCATCA
Inr
DPE
CTCATG
mDPE
D
MS2 intensity (AU)
WT
mTATA
mDPE
Time into nc14 (min)
E
Nucleus number
WT
mTATA
mDPE
MS2 intensity (AU)
Time into nc14 (min)
F
Output
Amplitude
Duration
Total RNA produced (AU)
MS2 intensity (AU)
Time (min)
1.8 x10-38
1.4 x10-107
1.1 x10-10
5.3 x10-84
2.8 x10-18
5.1 x10-7
% of WT 16 45
% of WT 35 82
% of WT 87 82
G
Frequency
Fraction
WT
mTATA
mDPE
Number of burst
H
Onset of first burst
Time (min)
3.0 x10-10
5.5 x10-12
% of WT 159 155
I
Rate of burst induction
Burst per minute
1.8 x10-16
7.9 x10-61
% of WT 40 75
WT mTATA mDPE

Figure S7

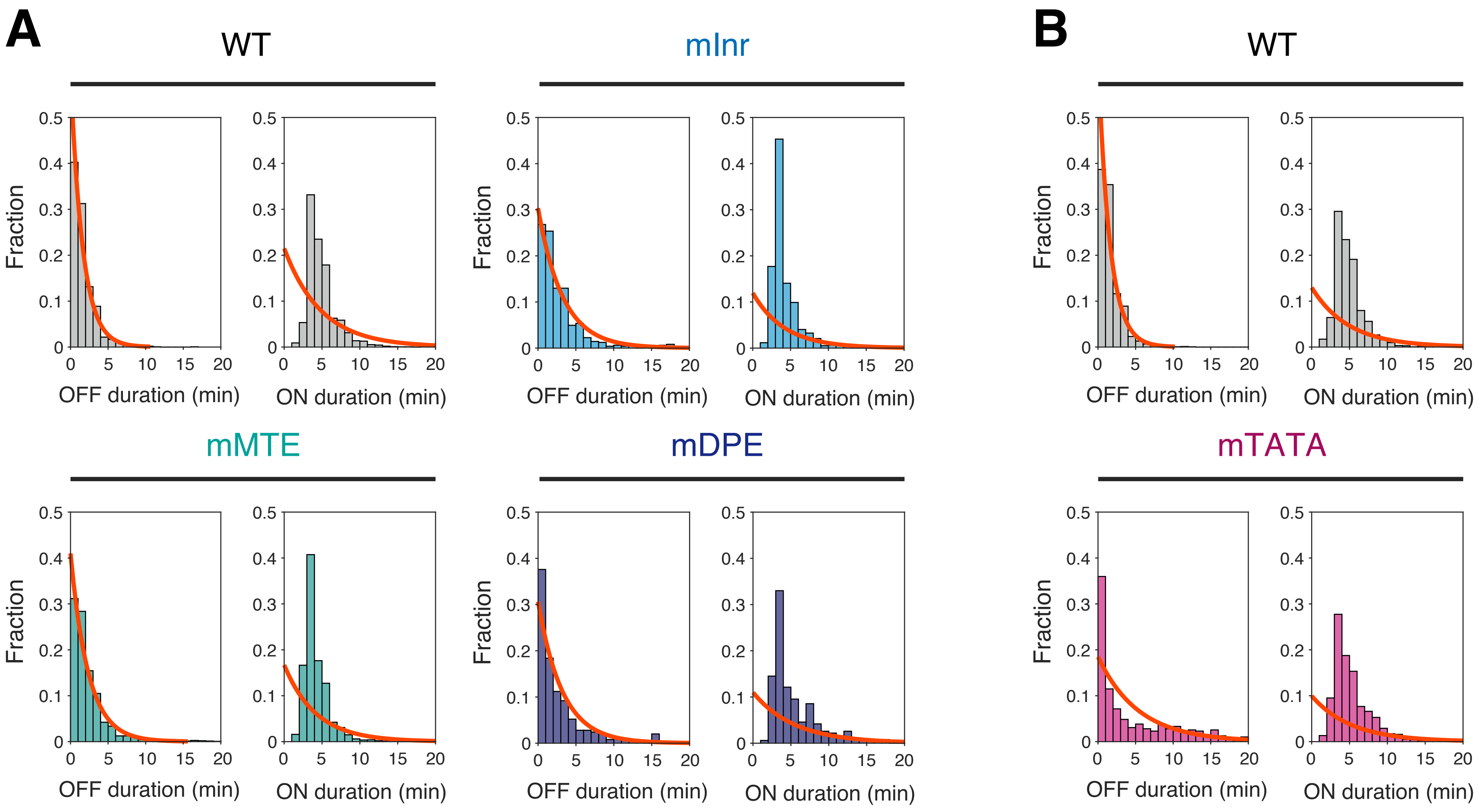

A
WT
mInr
mMTE
mDPE
B
WT
mTATA
Fraction
OFF duration (min)
ON duration (min)
0
0.1
0.2
0.3
0.4
0.5
5
10
15
20

Figure S8

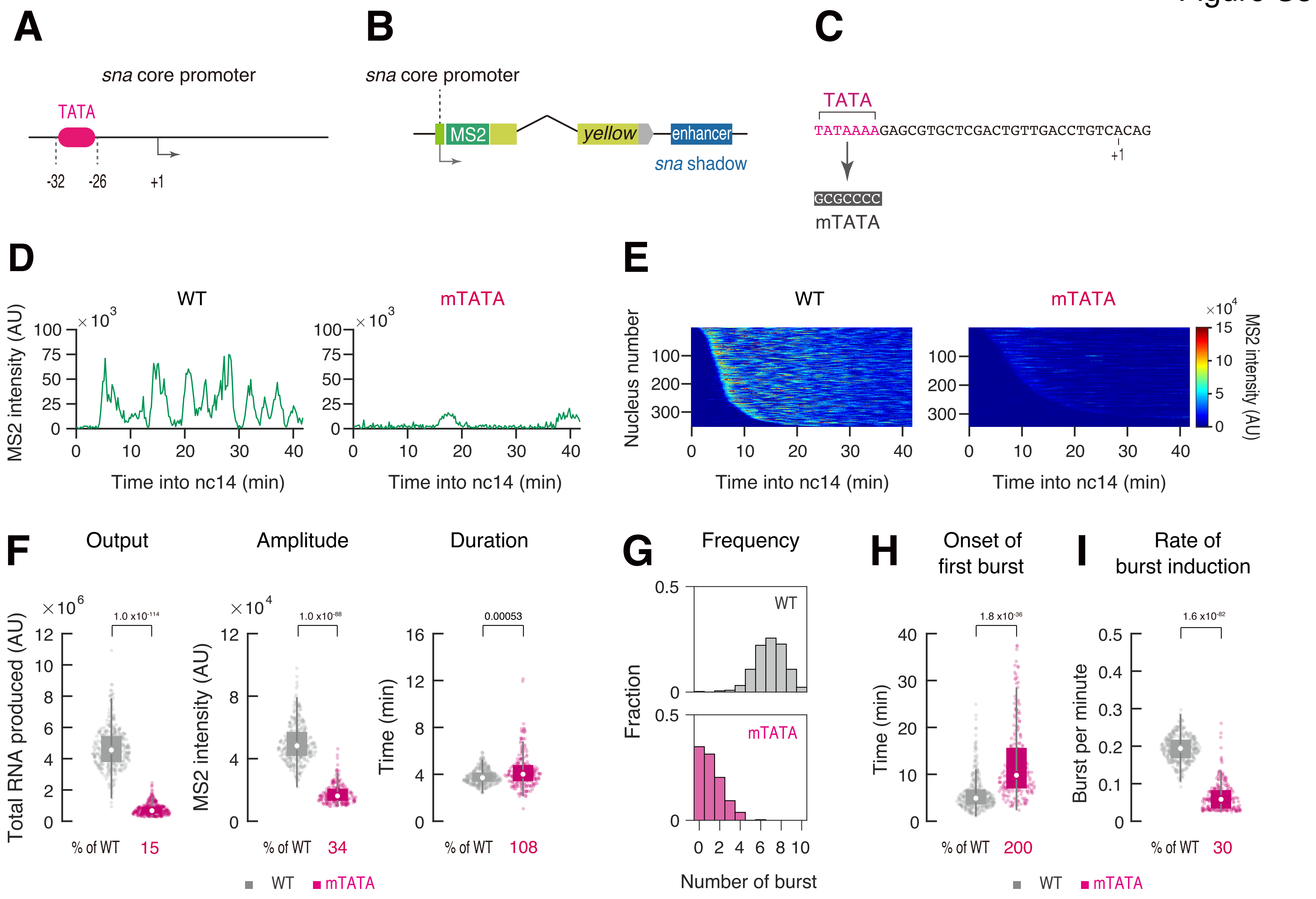
A
sna core promoter
TATA
-32
-26
+1
B
sna core promoter
MS2
yellow
enhancer
sna shadow
C
TATA
TATAAAAGAGCGTGCTCGACTGTTGACCTGTCACAG
+1
GCGCCCC
mTATA
D
WT
mTATA
MS2 intensity (AU)
Time into nc14 (min)
E
WT
mTATA
Nucleus number
Time into nc14 (min)
MS2 intensity (AU)
F
Output
Amplitude
Duration
Total RNA produced (AU)
MS2 intensity (AU)
Time (min)
1.0 x10-114
1.0 x10-88
0.00053
% of WT 15
% of WT 34
% of WT 108
G
Frequency
Fraction
WT
mTATA
Number of burst
H
Onset of first burst
Time (min)
1.8 x10-36
% of WT 200
I
Rate of burst induction
Burst per minute
1.6 x10-82
% of WT 30
WT
mTATA

Figure S9

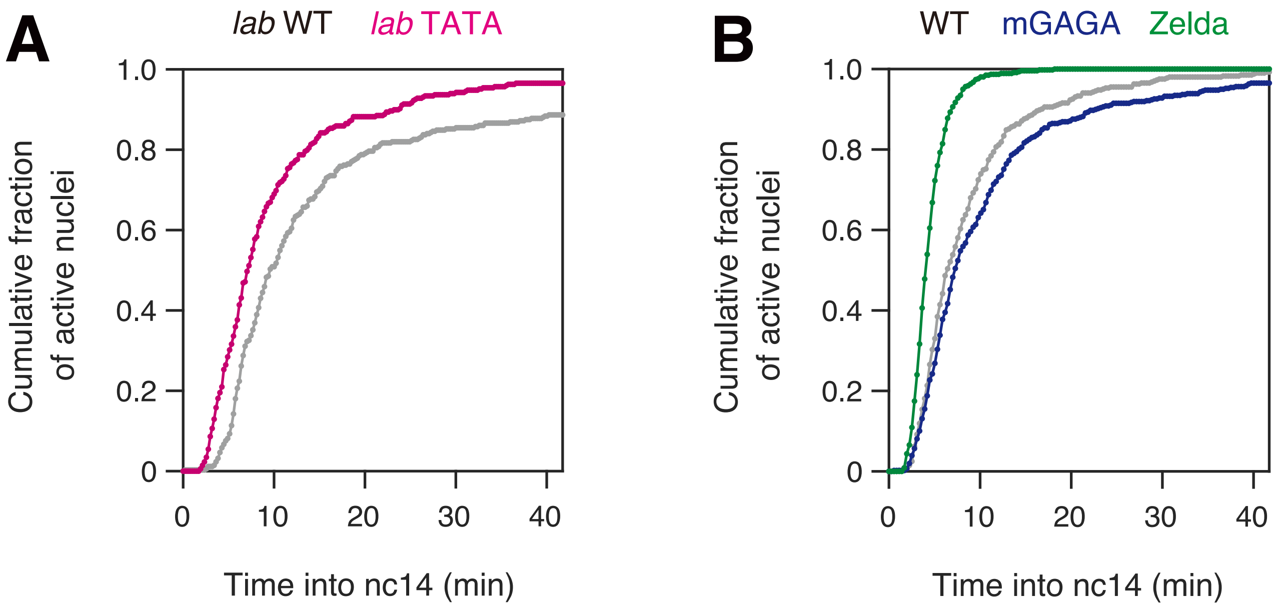
A
lab WT
lab TATA
1.0
0.8
0.6
0.4
0.2
0
Cumulative fraction of active nuclei
0
10
20
30
40
Time into nc14 (min)
B
WT
mGAGA
Zelda
1.0
0.8
0.6
0.4
0.2
0
Cumulative fraction of active nuclei
0
10
20
30
40
Time into nc14 (min)

Figure S10

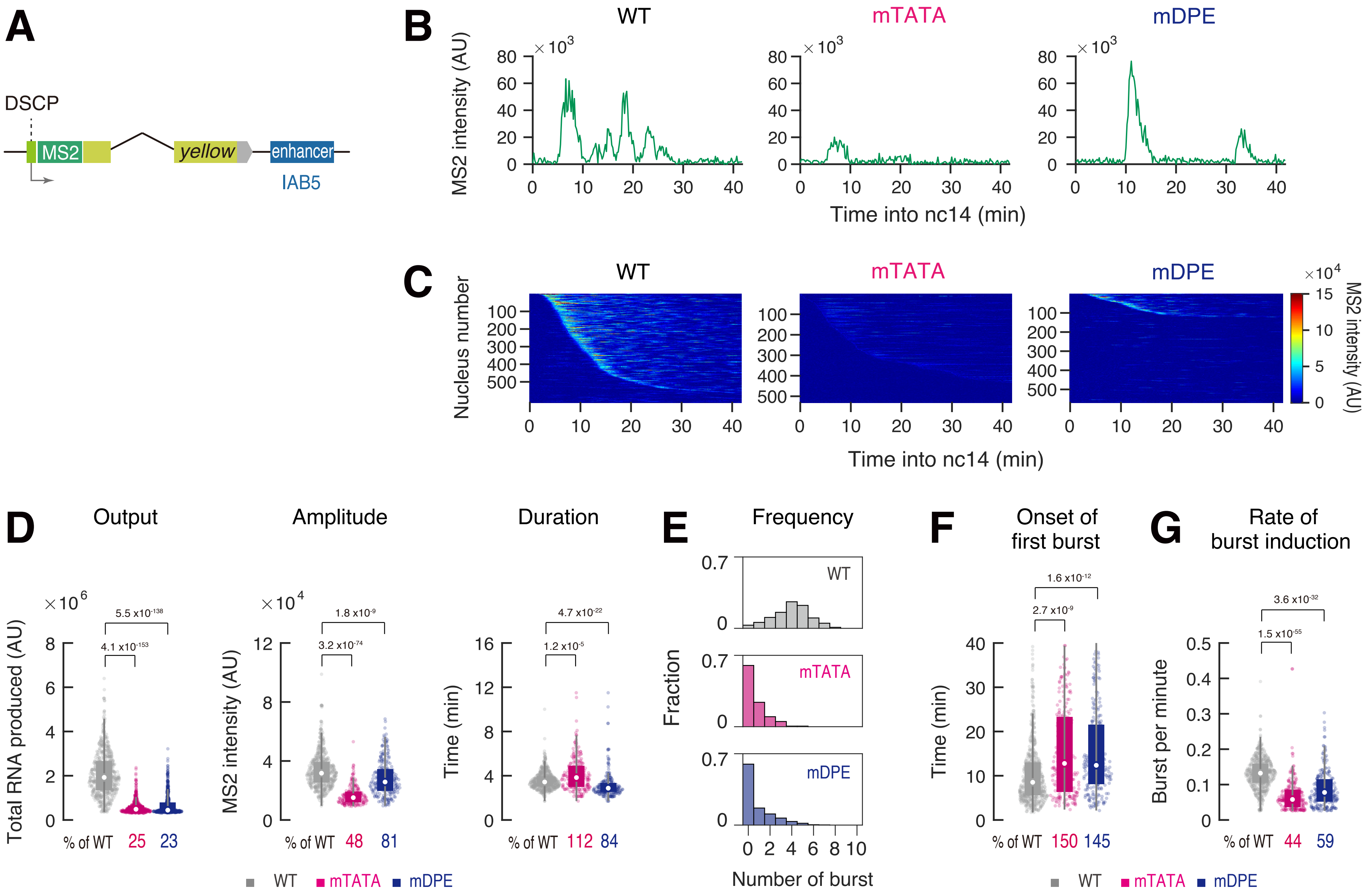

A
DSCP
MS2
yellow
enhancer
IAB5
B
WT
mTATA
mDPE
MS2 intensity (AU)
Time into nc14 (min)
C
WT
mTATA
mDPE
Nucleus number
MS2 intensity (AU)
Time into nc14 (min)
D
Output
Amplitude
Duration
Total RNA produced (AU)
MS2 intensity (AU)
Time (min)
% of WT 25 23
% of WT 48 81
% of WT 112 84
E
Frequency
Fraction
Number of burst
F
Onset of first burst
Time (min)
% of WT 150 145
G
Rate of burst induction
Burst per minute
% of WT 44 59
WT mTATA mDPE

Figure S11

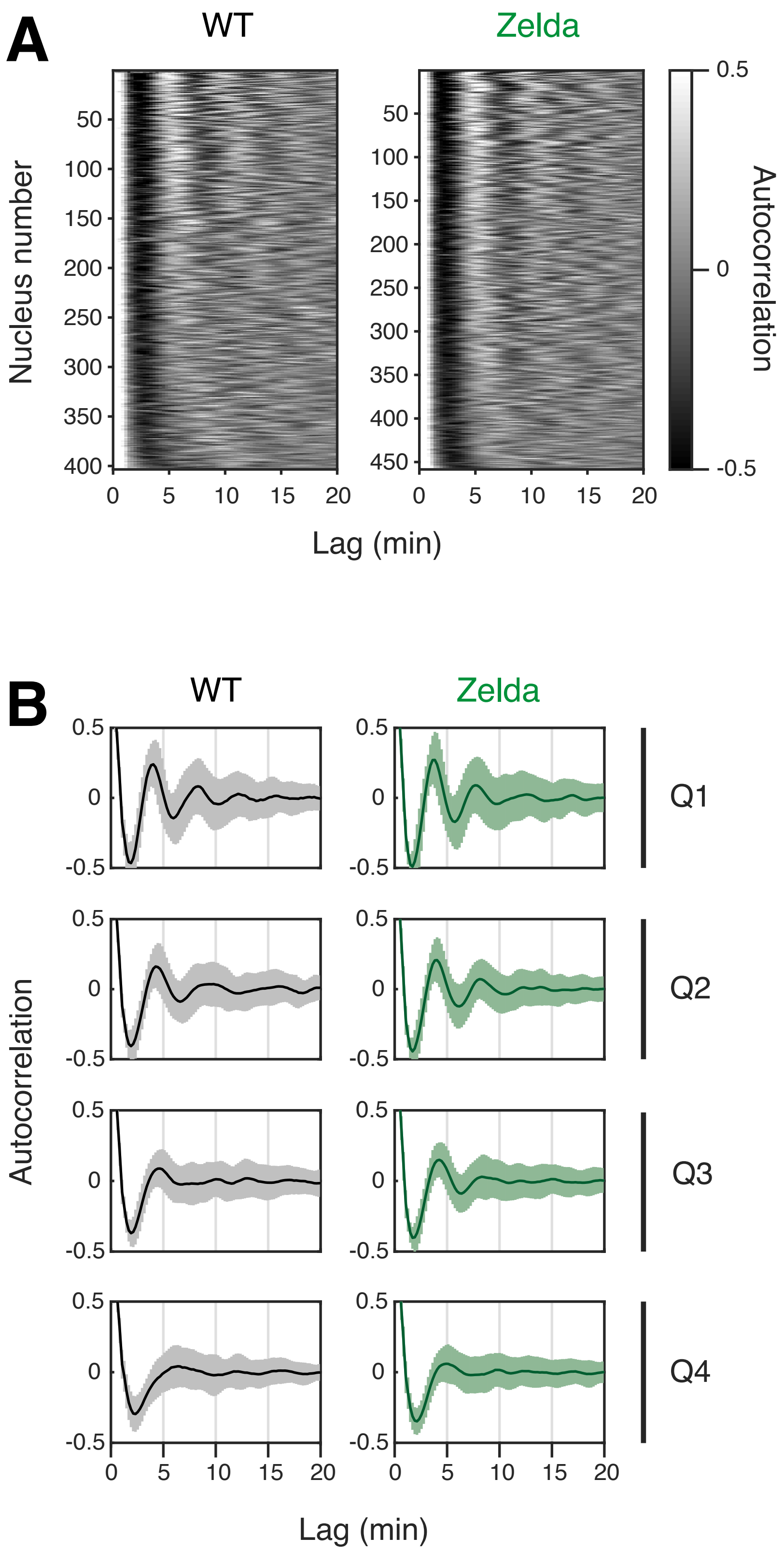
A
WT
Zelda
Nucleus number
50
100
150
200
250
300
350
400
450
0
5
10
15
20
Lag (min)
0.5
0
-0.5
Autocorrelation
B
WT
Zelda
Q1
Q2
Q3
Q4
Autocorrelation
Lag (min)

Figure S12

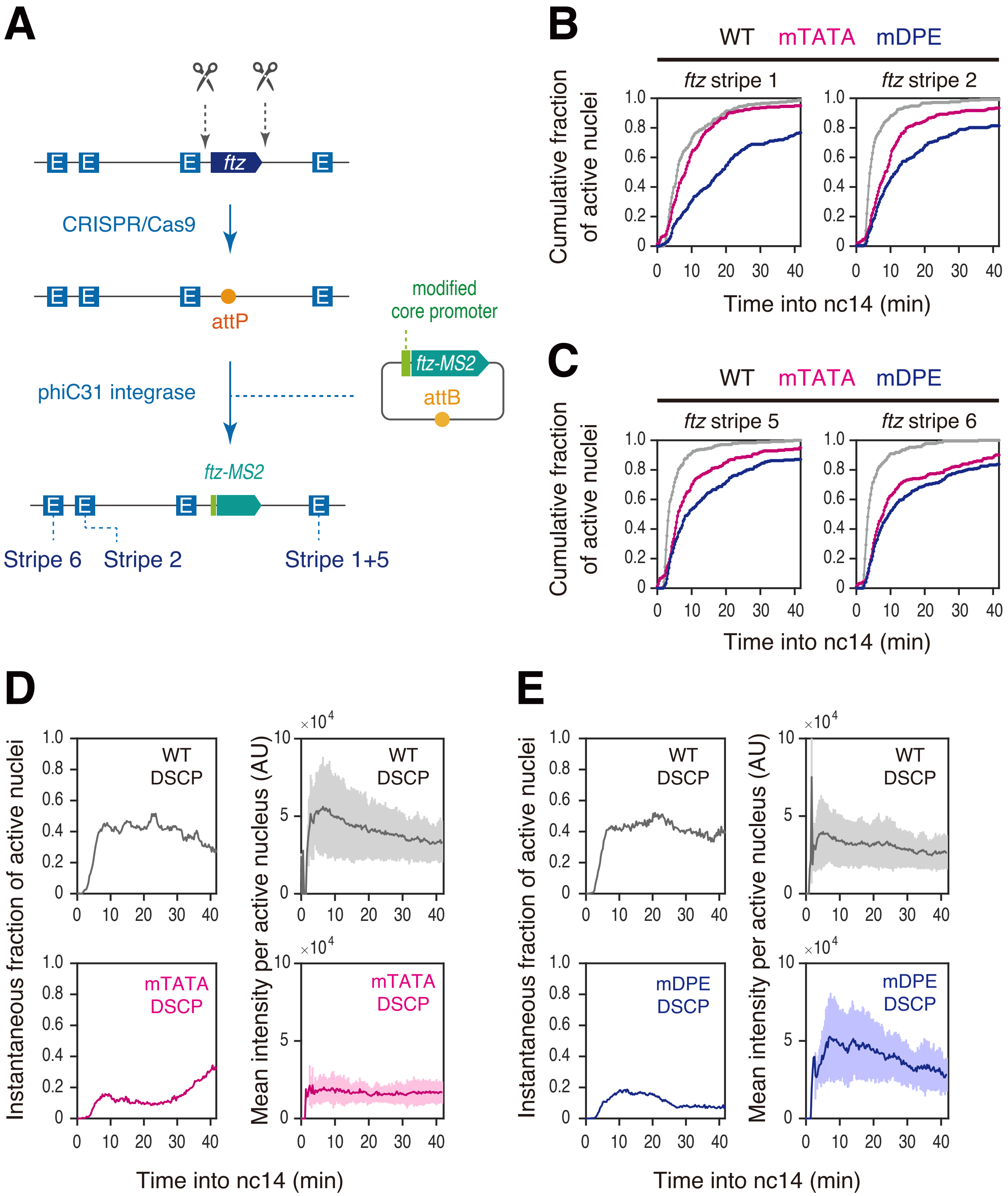

A
ftz
E
CRISPR/Cas9
attP
phiC31 integrase
modified
core promoter
ftz-MS2
attB
Stripe 6
Stripe 2
Stripe 1+5
B
WT mTATA mDPE
ftz stripe 1
ftz stripe 2
Cumulative fraction of active nuclei
Time into nc14 (min)
C
WT mTATA mDPE
ftz stripe 5
ftz stripe 6
Cumulative fraction of active nuclei
Time into nc14 (min)
D
WT DSCP
mTATA DSCP
Instantaneous fraction of active nuclei
Mean intensity per active nucleus (AU)
×10^4
Time into nc14 (min)
E
WT DSCP
mDPE DSCP
Instantaneous fraction of active nuclei
Mean intensity per active nucleus (AU)
×10^4
Time into nc14 (min)

Figure S13

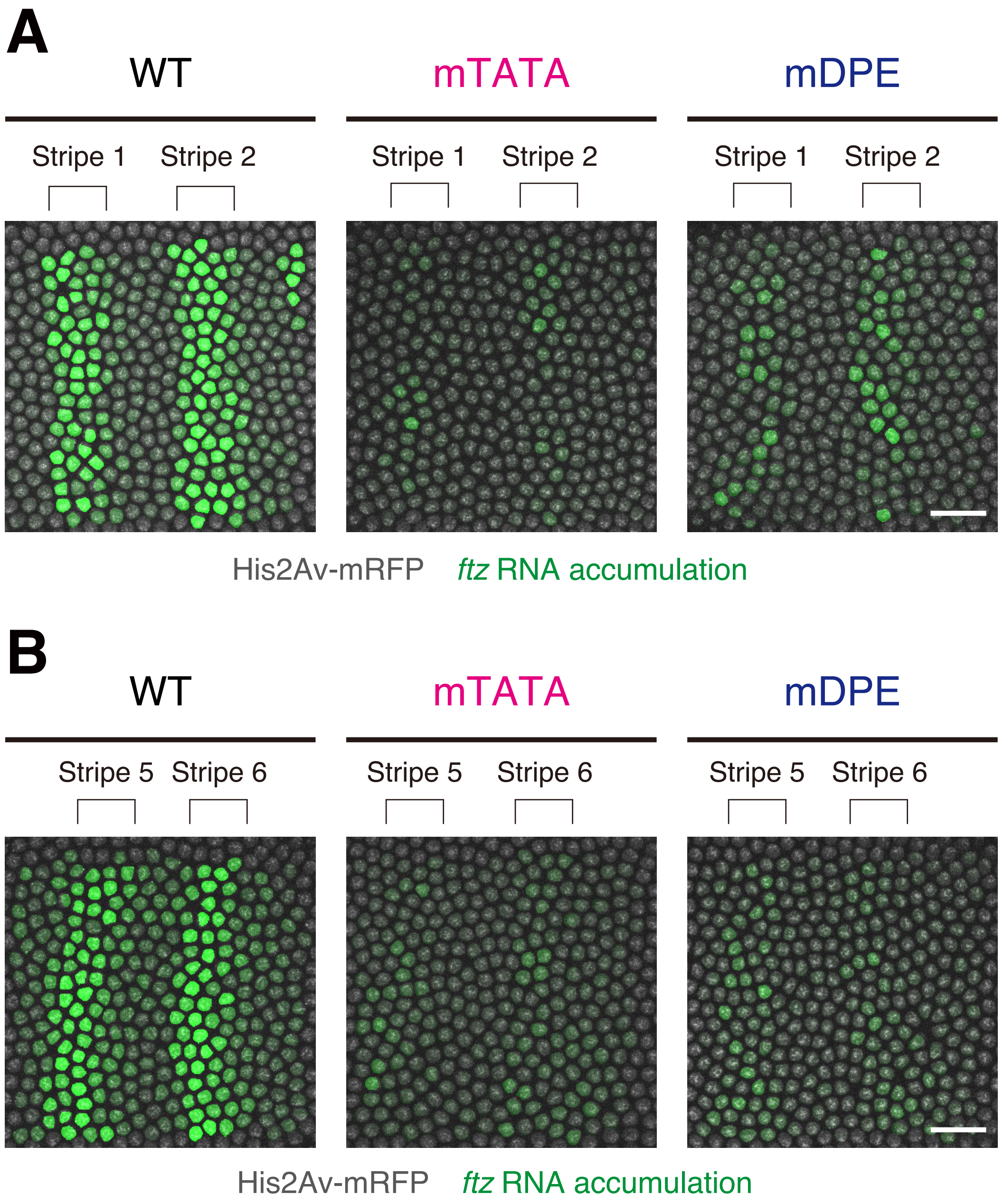
A
WT
mTATA
mDPE
Stripe 1
Stripe 2
Stripe 1
Stripe 2
Stripe 1
Stripe 2
His2Av-mRFP
ftz RNA accumulation
B
WT
mTATA
mDPE
Stripe 5
Stripe 6
Stripe 5
Stripe 6
Stripe 5
Stripe 6
His2Av-mRFP
ftz RNA accumulation

Figure S1. Visualization of enhancer-dependent transcription in living embryos.
(A) Overall structure of the *Drosophila* synthetic core promoter (DSCP).
(B) Schematic representation of the *yellow* reporter gene containing the 155-bp DSCP and 24x MS2 RNA stem loops within the 5´ UTR. Enhancer sequence was removed from the synthetic locus.
(C) Fluorescent *in situ* hybridization using probes against MS2 repeats and endogenous *sna*. Embryos at nc14 are shown. Images are cropped and rotated to align embryos (anterior to the left and posterior to the right). Scale bar indicates 50 μm.
(D) Representative trajectories of transcription activity of the MS2 reporter genes with (right) and without *sna* shadow enhancer (left) in individual nuclei. AU; arbitrary unit.
(E) MS2 trajectories for all analyzed nuclei. Each row represents MS2 trajectory for a single nucleus. A total of 372 and 371 ventral-most nuclei, respectively, were analyzed from three independent embryos for the reporter genes with (right) and without *sna* shadow enhancer (left). Nuclei were ordered by their onset of transcription in nc14. AU; arbitrary unit.

Figure S2. Quantification of transcriptional bursting.
(A) Snapshot of a gastrulating embryo (left) and false-coloring of ventral-most nuclei at mid nc14 (right). The maximum projected image of a histone marker (His2Av-mRFP) is shown in gray. Images are oriented with ventral view facing up. Scale bar indicates 20 μm.
(B) A representative image of raw MS2 fluorescence signal (left). A 11 x 11 pixels region with a single Z-plane was extracted from 3D volume data of the WT reporter shown in Figure 1. Surface plot represents raw intensities centering the brightest pixel (right).
(C) A 2D Gaussian fit to fluorescence intensities at the region shown in (B). Fitted results and analytical function are shown as a surface plot (left). X-Z projection of 2D Gaussian fit is shown in right. Each dot represents raw intensity, and trace represents fitted function.
(D) Trajectory of transcription activity shown in Figure 1D (left). Amplitude and duration were measured for each bursting event. Frequency was determined by counting total number of bursts during the analysis.

Figure S3. *gypsy* insertion reduces the frequency of transcriptional bursting.
(A) A 432-bp *gypsy* insulator was inserted between the *yellow* transcription unit and the *sna* shadow enhancer.
(B) Representative trajectories of transcription activity of the MS2 reporter genes with (right) and without *gypsy* insulator (left) in individual nuclei. AU; arbitrary unit.

(C) MS2 trajectories for all analyzed nuclei. Each row represents the MS2 trajectory for a single nucleus. A total of 371 and 380 ventral-most nuclei, respectively, were analyzed from three independent embryos for the reporter genes with (right) and without *gypsy* insulator (left). The data of the reporter gene without *gypsy* insulator is the same as the reporter gene containing *sna* shadow enhancer shown in Figure S1E. Nuclei were ordered by their onset of transcription in nc14. AU; arbitrary unit.

(D) Boxplots showing the distribution of total output (left), burst amplitude (middle) and burst duration (right). The box indicates the lower (25%) and upper (75%) quantile and the open circle indicates the median. Whiskers extend to the most extreme, non-outlier data points. A total of 371 and 380 ventral-most nuclei, respectively, were analyzed from three independent embryos for the reporter genes with and without *gypsy* insulator. Median values relative to the - *gypsy* reporter are shown at the bottom. The *p*-values of two-sided Wilcoxon rank sum test are shown at the top. The data of the reporter gene without *gypsy* insulator are the same as the WT reporter shown in Figure 5D. AU; arbitrary unit.

(E) Histograms showing the distribution of burst frequency. A total of 371 and 380 ventral-most nuclei, respectively, were analyzed from three independent embryos for the reporter genes with and without *gypsy* insulator. The data of the reporter gene without *gypsy* insulator is the same as the WT reporter shown in Figure 5E.

(F and G) Boxplots showing the distribution of the timing of first burst (F) and the burst frequency normalized by the length of time after the first burst (G). The box indicates the lower (25%) and upper (75%) quantile and the open circle indicates the median. Whiskers extend to the most extreme, non-outlier data points. A total of 371 and 380 ventral-most nuclei, respectively, were analyzed from three independent embryos for the reporter genes with and without *gypsy* insulator. Median values relative to the - *gypsy* reporter are shown at the bottom. The *p*-values of two-sided Wilcoxon rank sum test are shown at the top. The data of the reporter gene without *gypsy* insulator are the same as the WT reporter shown in Figure 5F and G.

Figure S4. Distribution of total output in three independent embryos.

(A) Boxplots showing the distribution of total output in each of three independent embryos. The box indicates the lower (25%) and upper (75%) quantile and the open circle indicates the median. Whiskers extend to the most extreme, non-outlier data points. A number of nuclei analyzed for each reporter gene is as follows. WT; 147, 129 and 127. mInr; 150, 145 and 140. mMTE; 156, 158 and 195. mDPE; 145, 135 and 164. AU; arbitrary unit.

(B) Cumulative fraction of actively transcribing nuclei at the ventral region. A total of 403, 435, 509 and 444 ventral-most nuclei were analyzed from three independent embryos for WT, mInr, mMTE and mDPE DSCP.
(C) Boxplots showing the distribution of total output in each of three independent embryos. The box indicates the lower (25%) and upper (75%) quantile and the open circle indicates the median. Whiskers extend to the most extreme, non-outlier data points. A number of nuclei analyzed for each reporter gene is as follows. WT; 133, 140 and 128. mTATA; 139, 139 and 128. AU; arbitrary unit.
(D) Cumulative fraction of actively transcribing nuclei at the ventral region. A total of 401 and 406 ventral-most nuclei were analyzed from three independent embryos for WT and mTATA DSCP.
(E) Histograms showing the distribution of burst duration with (right) and without filtering (left). In total, 2009 independent bursts were extracted and analyzed using dataset of the WT DSCP reporter shown in Figure 1. Only a very small population (less than 2.5% of entire bursts) of transient signals were removed by filtering (see Materials and Methods).
(F) Boxplots showing the distribution of burst duration quantified from the analysis of the reporter genes shown in Figure 1 and 2 without filtering. The box indicates the lower (25%) and upper (75%) quantile and the open circle indicates the median. Whiskers extend to the most extreme, non-outlier data points. Median values relative to the WT DSCP reporter are shown at the bottom.

Figure S5. Analysis of DSCP reporters under the control of *rho*NEE.
(A) Schematic representation of the *yellow* reporter gene containing the 155-bp DSCP, the 666-bp *rho*NEE, and 24x MS2 RNA stem loops within the 5´ UTR.
(B) Representative trajectories of transcription activity of the MS2 reporter genes with WT (left), mTATA (left middle), mInr (middle), mMTE (right middle) and mDPE DSCP (right) in individual nuclei. AU; arbitrary unit.
(C) MS2 trajectories for all analyzed nuclei. Each row represents the MS2 trajectory for a single nucleus. A total of 213, 211, 224, 216 and 210 nuclei located at the expression domain, respectively, were analyzed from three independent embryos for the reporter genes with WT (left), mTATA (left middle), mInr (middle), mMTE (right middle) and mDPE DSCP (right). Nuclei were ordered by their onset of transcription in nc14. AU; arbitrary unit.
(D) Cumulative fraction of actively transcribing nuclei at the ventral neurogenic ectoderm. A total of 213, 211, 224, 216 and 210 nuclei located at the expression domain, respectively,

were analyzed from three independent embryos for the reporter genes with WT, mTATA, mInr, mMTE and mDPE DSCP.

(E) Boxplots showing the distribution of total output (left), burst amplitude (middle) and burst duration (right). The box indicates the lower (25%) and upper (75%) quantile and the open circle indicates the median. Whiskers extend to the most extreme, non-outlier data points. A total of 213, 211, 224, 216 and 210 nuclei located at the expression domain, respectively, were analyzed from three independent embryos for the reporter genes with WT, mTATA, mInr, mMTE and mDPE DSCP. Median values relative to the WT reporter are shown at the bottom. The *p*-values of two-sided Wilcoxon rank sum test are shown at the top. AU; arbitrary unit.

(F) Histograms showing the distribution of burst frequency. A total of 213, 211, 224, 216 and 210 nuclei located at the expression domain, respectively, were analyzed from three independent embryos for the reporter genes with WT (top), mTATA (upper middle), mINR (middle), mMTE (lower middle) and mDPE DSCP (bottom).

(G and H) Boxplots showing the distribution of the timing of first burst (G) and the burst frequency normalized by the length of time after the first burst (H). The box indicates the lower (25%) and upper (75%) quantile and the open circle indicates the median. Whiskers extend to the most extreme, non-outlier data points. A total of 213, 211, 224, 216 and 210 nuclei located at the expression domain, respectively, were analyzed from three independent embryos for the reporter genes with WT, mTATA, mInr, mMTE and mDPE DSCP. Median values relative to the WT reporter are shown at the bottom. The *p*-values of two-sided Wilcoxon rank sum test are shown at the top.

Figure S6. Analysis of the *rho* core promoter linked to the *rho*NEE.

(A) Endogenous *rho* core promoter contains TATA, Inr and DPE.

(B) Schematic representation of the *yellow* reporter gene containing the minimal 100-bp endogenous *rho* core promoter, the 666-bp *rho*NEE, and 24x MS2 RNA stem loops within the 5′ UTR. The *rho*NEE was placed ~1.5 kb upstream of the core promoter.

(C) TATA and DPE were mutated as indicated.

(D) Representative trajectories of transcription activity of the MS2 reporter genes with WT (left), mTATA (middle) and mDPE (right) in individual nuclei. AU; arbitrary unit.

(E) MS2 trajectories for all analyzed nuclei. Each row represents the MS2 trajectory for a single nucleus. A total of 377, 376 and 330 nuclei located at the expression domain, respectively, were analyzed from three independent embryos for the reporter genes with WT (left), mTATA (middle) and mDPE (right). Nuclei were ordered by their onset of transcription in nc14. AU; arbitrary unit.

(F) Boxplots showing the distribution of total output (left), burst amplitude (middle) and burst duration (right). The box indicates the lower (25%) and upper (75%) quantile and the open circle indicates the median. Whiskers extend to the most extreme, non-outlier data points. A total of 377, 376 and 330 nuclei located at the expression domain, respectively, were analyzed from three independent embryos for the reporter genes with WT, mTATA and mDPE. Median values relative to the WT reporter are shown at the bottom. The *p*-values of two-sided Wilcoxon rank sum test are shown at the top. AU; arbitrary unit.

(G) Histograms showing the distribution of burst frequency. A total of 377, 376 and 330 nuclei located at the expression domain, respectively, were analyzed from three independent embryos for the reporter genes with WT (top), mTATA (middle) and mDPE (bottom).

(H and I) Boxplots showing the distribution of the timing of first burst (H) and the burst frequency normalized by the length of time after the first burst (I). The box indicates the lower (25%) and upper (75%) quantile and the open circle indicates the median. Whiskers extend to the most extreme, non-outlier data points. A total of 377, 376 and 330 nuclei located at the expression domain, respectively, were analyzed from three independent embryos for the reporter genes with WT, mTATA and mDPE. Median values relative to the WT reporter are shown at the bottom. The *p*-values of two-sided Wilcoxon rank sum test are shown at the top.

Figure S7. Distribution of OFF and ON duration.

(A and B) Histograms showing the distribution of OFF and ON duration of each discrete burst. Datasets of the reporter genes shown in Figure 1 and 2 were used for the analysis. Red solid line indicates fitted curve to an exponential distribution. A total number of OFF and ON events analyzed for each reporter is as follows. (A) WT; 1592 and 2009. mInr; 383 and 647. mMTE; 1304 and 1770. mDPE; 195 and 414. (B) WT; 1645 and 2063. mTATA; 208 and 475.

Figure S8. Analysis of the *sna* core promoter linked to the *sna* shadow enhancer.

(A) Endogenous *sna* core promoter contains TATA.

(B) Schematic representation of the *yellow* reporter gene containing the minimal 100-bp endogenous *sna* core promoter, the 1.5-kb *sna* shadow enhancer, and 24x MS2 RNA stem loops within the 5′ UTR. The *sna* shadow enhancer was placed ~6.5 kb downstream of the core promoter.

(C) TATA was mutated as indicated.

(D) Representative trajectories of transcription activity of the MS2 reporter genes with WT (left) and mTATA (right) in individual nuclei. AU; arbitrary unit.
(E) MS2 trajectories for all analyzed nuclei. Each row represents the MS2 trajectory for a single nucleus. A total of 352 and 344 ventral-most nuclei, respectively, were analyzed from three independent embryos for the reporter genes with WT (left) and mTATA (right). Nuclei were ordered by their onset of transcription in nc14. AU; arbitrary unit.
(F) Boxplots showing the distribution of total output (left), burst amplitude (middle) and burst duration (right). The box indicates the lower (25%) and upper (75%) quantile and the open circle indicates the median. Whiskers extend to the most extreme, non-outlier data points. A total of 352 and 344 ventral-most nuclei, respectively, were analyzed from three independent embryos for the reporter genes with WT and mTATA. Median values relative to the WT reporter are shown at the bottom. The p-values of two-sided Wilcoxon rank sum test are shown at the top. AU; arbitrary unit.
(G) Histograms showing the distribution of burst frequency. A total of 352 and 344 ventral-most nuclei, respectively, were analyzed from three independent embryos for the reporter genes with WT (top) and mTATA (bottom).
(H and I) Boxplots showing the distribution of the timing of first burst (H) and the burst frequency normalized by the length of time after the first burst (I). The box indicates the lower (25%) and upper (75%) quantile and the open circle indicates the median. Whiskers extend to the most extreme, non-outlier data points. A total of 352 and 344 ventral-most nuclei, respectively, were analyzed from three independent embryos for the reporter genes with WT and mTATA. Median values relative to the WT reporter are shown at the bottom. The p-values of two-sided Wilcoxon rank sum test are shown at the top.

Figure S9. TATA and Zelda shorten induction time of transcription.
(A) Cumulative fraction of actively transcribing nuclei at the ventral region. A total of 344 and 348 ventral-most nuclei were analyzed from three independent embryos for the reporter genes with unmodified TATA-less and modified TATA-containing *lab* core promoter.
(B) Cumulative fraction of actively transcribing nuclei at the ventral region. A total of 403, 458 and 458 ventral-most nuclei were analyzed from three independent embryos for WT, mGAGA and Zelda DSCP. Plot of WT is the same as the plot in Figure S4B.

Figure S10. Analysis of DSCP reporters under the control of IAB5 enhancer.
(A) Schematic representation of the *yellow* reporter gene containing the 155-bp DSCP, the 1013-bp IAB5 enhancer, and 24x MS2 RNA stem loops within the 5′ UTR.

(B) Representative trajectories of transcription activity of the MS2 reporter genes with WT (left), mTATA (middle) and mDPE (right) in individual nuclei. AU; arbitrary unit.
(C) MS2 trajectories for all analyzed nuclei. Each row represents the MS2 trajectory for a single nucleus. A total of 618, 531 and 569 nuclei located at the expression domain, respectively, were analyzed from three independent embryos for the reporter genes with WT (left), mTATA (middle) and nDPE (right). Nuclei were ordered by their onset of transcription in nc14. AU; arbitrary unit.
(D) Boxplots showing the distribution of total output (left), burst amplitude (middle) and burst duration (right). The box indicates the lower (25%) and upper (75%) quantile and the open circle indicates the median. Whiskers extend to the most extreme, non-outlier data points. A total of 618, 531 and 569 nuclei located at the expression domain, respectively, were analyzed from three independent embryos for the reporter genes with WT, mTATA and mDPE. Median values relative to the WT reporter are shown at the bottom. The *p*-values of two-sided Wilcoxon rank sum test are shown at the top. AU; arbitrary unit.
(E) Histograms showing the distribution of burst frequency. A total of 618, 531 and 569 nuclei located at the expression domain, respectively, were analyzed from three independent embryos for the reporter genes with WT (top), mTATA (middle) and mDPE (bottom).
(F and G) Boxplots showing the distribution of the timing of first burst (F) and the burst frequency normalized by the length of time after the first burst (G). The box indicates the lower (25%) and upper (75%) quantile and the open circle indicates the median. Whiskers extend to the most extreme, non-outlier data points. A total of 618, 531 and 569 nuclei located at the expression domain, respectively, were analyzed from three independent embryos for the reporter genes with WT, mTATA and mDPE. Median values relative to the WT reporter are shown at the bottom. The *p*-values of two-sided Wilcoxon rank sum test are shown at the top.

Figure S11. Autocorrelation analysis of the Zelda reporter gene.
(A) Autocorrelation values obtained from the analysis of MS2 trajectories of the reporter genes containing WT and Zelda DSCP are plotted as a heatmap. Each row represents autocorrelation values calculated from individual nuclei. Datasets of the reporter genes shown in Figure 4 were used for the analysis.
(B) Nuclei were divided into Q1, Q2, Q3 and Q4 quartile from the descending order in (A). Solid line indicates mean autocorrelation value, and shade represents standard deviation of the mean in each subgroup.

Figure S12. Dynamics of *ftz* transcription at the engineered alleles.

(A) Schematic representation of the core promoter modification approach. Approximate locations of enhancers regulating *ftz* expression in early embryos are indicated as "E". Note that extra sequences derived from plasmid backbone, ampicillin resistant gene and mini-white marker gene were incorporated into the *ftz* locus after phiC31-mediated integration.

(B) Cumulative fraction of actively transcribing nuclei at anterior stripe 1/2. A total of 234, 236 and 227 nuclei at stripe 1, and 248, 241 and 242 nuclei at stripe 2 were analyzed from three independent embryos for the *ftz-MS2* with WT, mTATA and mDPE core promoter, respectively.

(C) Cumulative fraction of actively transcribing nuclei at posterior stripe 5/6. A total of 243,238 and 240 nuclei at stripe 5, and 232, 239 and 240 nuclei at stripe 6 were analyzed from three independent embryos for the *ftz-MS2* with WT, mTATA and mDPE core promoter, respectively.

(D) Instantaneous fraction of actively transcribing nuclei (left) and mean MS2 intensity per actively transcribing nucleus (right). A total of 401 and 406 ventral-most nuclei, respectively, were analyzed from three independent embryos for the reporter genes with WT and mTATA DSCP shown in Figure 2. AU; arbitrary unit. Shades represent the standard deviation of the mean across active nuclei at a given time.

(E) Instantaneous fraction of actively transcribing nuclei (left) and mean MS2 intensity per actively transcribing nucleus (right). A total of 403 and 444 ventral-most nuclei, respectively, were analyzed from three independent embryos for the reporter genes with WT and mDPE DSCP shown in Figure 1. AU; arbitrary unit. Shades represent the standard deviation of the mean across active nuclei at a given time.

Figure S13. TATA and DPE mutations lead to sporadic stripe patterns.

(A) Computational reconstitution of *ftz* mRNA accumulation at the anterior stripe region. Scale bar indicates 20 μm.

(B) Computational reconstitution of *ftz* mRNA accumulation at the posterior stripe region. Scale bar indicates 20 μm.

Movie Legends

Movie S1. Live imaging of the MS2 reporters with WT, mInr, mMTE and mDPE DSCP.

Live imaging of *MS2-yellow-sna shadow enhancer* containing WT (left), mInr (left middle), mMTE (right middle) and mDPE DSCP (right) during nc14. The maximum projected images of MCP-GFP and His2Av-mRFP are shown in green and red, respectively. Images are oriented with ventral view facing up.

Movie S2. Live imaging of the MS2 reporters with WT and mTATA DSCP.

Live imaging of *MS2-yellow-sna shadow enhancer* containing WT (left) and mTATA DSCP (right) during nc14. The maximum projected images of MCP-GFP and His2Av-mRFP are shown in green and red, respectively. Images are oriented with ventral view facing up.

Movie S3. Live imaging of the MS2 reporter with WT and TATA *lab* core promoter.

Live imaging of *MS2-yellow-sna shadow enhancer* containing unmodified TATA-less (left) and modified TATA-containing *lab* core promoter (right) during nc14. The maximum projected images of MCP-GFP and His2Av-mRFP are shown in green and red, respectively. Images are oriented with ventral view facing up.

Movie S4. Live imaging of the MS2 reporters with WT, mGAGA and Zelda DSCP.

Live imaging of *MS2-yellow-sna shadow enhancer* containing WT (left), mGAGA (middle) and Zelda DSCP (right) during nc14. The maximum projected images of MCP-GFP and His2Av-mRFP are shown in green and red, respectively. Images are oriented with ventral view facing up.

Movie S5. Live imaging of the MS2 reporters with mTATA and Zelda/mTATA DSCP.

Live imaging of *MS2-yellow-sna shadow enhancer* containing mTATA (left) and Zelda/mTATA DSCP (right) during nc14. The maximum projected images of MCP-GFP and His2Av-mRFP are shown in green and red, respectively. Images are oriented with ventral view facing up.

Movie S6. Live imaging of *ftz-MS2* complementation alleles.

Live imaging of *ftz-MS2* containing WT (top), mTATA (middle) and mDPE core promoter

(bottom) during nc14. Anterior stripe 1 and 2 regions were shown in left. Posterior stripe 5 and 6 regions were shown in right. The maximum projected images of MCP-GFP and His2Av-mRFP are shown in green and red, respectively. Images are oriented with anterior to the left.
