# Supplementary material for: Dynamic modulation of enhancer responsiveness by core promoter elements in living Drosophila embryos
Source: Nucleic Acids Res. 2021 Dec 13;50(1):92–107. doi: 10.1093/nar/gkab1177 (PMC8754644; doi:10.1093/nar/gkab1177)
Supplement: gkab1177_Supplemental_Files [file gkab1177_supplemental_files.zip › Supplementary_Methods.pdf]

Supplementary Materials and Methods

Plasmid construction

pbphi-DSCP$_{mTATA}$-MS2-yellow-sna shadow enhancer

Two DNA oligos (5′-TTT CCC TCG AGG AGC TCG CCC GGG GAT CGA GCG CAG CGG GCG CCC CGG GCG CGG GGT GGC TGA GAG CAT CAG TTG TGA ATG AAT GTT CGA GCC GAG C-3′) and (5′-GGA AAG GAT CCG TTT GGT ATG CGT CTT GTG ATT CAA AGT TGG CTT ATT CAA AGG ATA TTA ACG AAG GCA GCG GCA CGT CTG CTC GGC TCG AAC AT-3′) were annealed and blunt-ended by PCR. Resulting DNA fragment was inserted between XhoI and BamHI sites of pbphi-DSCP-MS2-yellow (1) after digestion with corresponding restriction enzymes. Subsequently, a DNA fragment containing *sna* shadow enhancer was purified from pbphi-snail shadow enhancer (2) by digesting with HindIII and NheI. The resulting fragment was inserted between HindIII and NheI sites of the plasmid.

pbphi-DSCP$_{mInr}$-MS2-yellow-sna shadow enhancer

Two DNA oligos (5′-TTT CCC TCG AGG AGC TCG CCC GGG GAT CGA GCG CAG CGG TAT AAA AGG GCG CGG GGT GGC TGA GAG CAG TGA CAG TGA ATG AAT GTT CGA GCC GAG C-3′) and (5′-GGA AAG GAT CCG TTT GGT ATG CGT CTT GTG ATT CAA AGT TGG CTT ATT CAA AGG ATA TTA ACG AAG GCA GCG GCA CGT CTG CTC GGC TCG AAC AT-3′) were annealed and blunt-ended by PCR. Resulting DNA fragment was inserted between XhoI and BamHI sites of pbphi-DSCP-MS2-yellow (1) after digestion with corresponding restriction enzymes. Subsequently, a DNA fragment containing *sna* shadow enhancer was inserted between HindIII and NheI sites of the plasmid.

pbphi-DSCP$_{mMTE}$-MS2-yellow-sna shadow enhancer

Two DNA oligos (5′-TTT CCC TCG AGG AGC TCG CCC GGG GAT CGA GCG CAG CGG TAT AAA AGG GCG CGG GGT GGC TGA GAG CAT CAG TTG TGA ATG AAT GTT ATC CAC GAG C-3′) and (5′-GGA AAG GAT CCG TTT GGT ATG CGT CTT GTG ATT CAA AGT TGG CTT ATT CAA AGG ATA TTA ACG AAG GCA GCG GCA CGT CTG CTC GTG GAT AAC AT-3′) were annealed and blunt-ended by PCR. Resulting DNA fragment was inserted between XhoI and BamHI sites of pbphi-DSCP-MS2-yellow (1) after digestion with corresponding restriction enzymes. Subsequently, a DNA fragment containing *sna* shadow enhancer was inserted between HindIII and NheI sites of the plasmid.

pbphi-DSCP$_{mDPE}$-MS2-yellow-sna shadow enhancer

Two DNA oligos (5′-TTA AAC TCG AGG AGC TCG CCC GGG GAT CGA GCG CAG CGG TAT AAA AGG GCG CGG GGT GGC TGA GAG CAT CAG TTG TGA ATG AAT GTT CGA GCC GAG C-3′) and (5′-ACA TGG GAT CCG TTT GGT ATG CGT CTT GTG ATT CAA AGT TGG CTT ATT CAA AGG ATA TTA ACG AAG GCA GCG GCC ATG AGG CTC GGC TCG AAC ATT CAT T-3′) were annealed and blunt-ended by PCR. Resulting DNA fragment was inserted between XhoI and BamHI sites of pbphi-DSCP-MS2-yellow (1) after digestion with corresponding restriction enzymes. Subsequently, a DNA fragment containing *sna* shadow enhancer was inserted between HindIII and NheI sites of the plasmid.

pbphi-DSCP$_{mGAGA}$-MS2-yellow-sna shadow enhancer

Two DNA oligos (5′-TTA AAC TCG AGG AGC TCG CCC GGG GAT CGA GCG CAG CGG TAT AAA AGG GCG CGG GGT GGC TCA CTG CAT CAG TTG TGA ATG AAT GTT CGA GCC GAG C-3′) and (5′-ACA TGG GAT CCG TTT GGT ATG CGT CTT GTG ATT CAA AGT TGG CTT ATT CAA AGG ATA TTA ACG AAG GCA GCG GCA CGT CTG CTC GGC TCG AAC ATT CAT T-3′) were annealed and blunt-ended by PCR. Resulting DNA fragment was inserted between XhoI and BamHI sites of pbphi-DSCP-MS2-yellow (1) after digestion with corresponding restriction enzymes. Subsequently, a DNA fragment containing *sna* shadow enhancer was inserted between HindIII and NheI sites of the plasmid.

pbphi-DSCP$_{3xZelda}$-MS2-yellow-sna shadow enhancer

Two DNA oligos (5′-TTT CCC TCG AGC AGG TAG CCC GGG GAT CGA GCG CAG CGG TAT AAA AGG GCG CGG GGT GGC TGA GAG CAT CAG TTG TGA ATG AAT GTT CGA GCC GAG C-3′) and (5′-GGA AAG GAT CCG TTT GGT ATG CGT CTT GTG ATT CAA AGT TGG CTT ATT CAA AGG ATA TTA ACG AAG GCA GCG GCA CGT CTG CTC GGC TCG AAC AT-3′) were annealed and blunt-ended by PCR. Resulting DNA fragment was inserted between XhoI and BamHI sites of pbphi-DSCP-MS2-yellow (1) after digestion with corresponding restriction enzymes. Subsequently, two DNA oligos (5′-GGC CGC CAG GTA GCA GGT AGC-3′) and (5′-TCG AGC TAC CTG CTA CCT GGC-3′) were annealed and inserted into the plasmid using NotI and XhoI sites. Then, a DNA fragment containing *sna* shadow enhancer was inserted between HindIII and NheI sites of the plasmid.

labPr$_{WT}$-MS2-yellow-sna shadow enhancer

Two DNA oligos (5′-TTA AAG CGG CCG CGT CTG CAG AGG GGC GTG GCC AAG ACC AGC GGT TGT GCG GTC TGA AAG AAA CCG GGT TCG GGC CAG TAA TCA GTC AC-3′) and (5′-ACA TGG GAT CCC CGA AAA ACA CGA CTC CCG TTG GCG ATG ACG ACG ACG ACG TGC TGC CTG CGC GCT TAC CAA GTC GTG ACT GAT TAC TGG CCC GA-3′) were annealed and blunt-ended by PCR. Resulting DNA fragment was inserted between NotI and BamHI sites of pbphi-DSCP-MS2-yellow-sna shadow enhancer (1) after digestion with corresponding restriction enzymes.

labPr$_{TATA}$-MS2-yellow-sna shadow enhancer

Two DNA oligos (5′-TTA AAG CGG CCG CGT CTG CAG AGG GGC GTG GCC AAG ACC AGC GGT TGT GCG GTA TAA AAG AAA CCG GGT TCG GGC CAG TAA TCA GTC AC-3′) and (5′-ACA TGG GAT CCC CGA AAA ACA CGA CTC CCG TTG GCG ATG ACG ACG ACG ACG TGC TGC CTG CGC GCT TAC CAA GTC GTG ACT GAT TAC TGG CCC GA-3′) were annealed and blunt-ended by PCR. Resulting DNA fragment was inserted between NotI and BamHI sites of pbphi-DSCP-MS2-yellow-sna shadow enhancer (1) after digestion with corresponding restriction enzymes.

pbphi-DSCP$_{WT}$-MS2-yellow-rhoNEE

A DNA fragment containing *rho*NEE was inserted between HindIII and NheI sites of pbphi-DSCP-MS2-yellow-sna shadow enhancer (1). Sequence of *rho*NEE is the same as one used in the previous study (3).

pbphi-DSCP$_{mTATA}$-MS2-yellow-rhoNEE

A DNA fragment containing *rho*NEE was inserted between HindIII and NheI sites of pbphi-DSCP$_{mTATA}$-MS2-yellow-sna shadow enhancer. Sequence of *rho*NEE is the same as one used in the previous study (3).

pbphi-DSCP$_{mInr}$-MS2-yellow-rhoNEE

A DNA fragment containing *rho*NEE was inserted between HindIII and NheI sites of pbphi-DSCP$_{mInr}$-MS2-yellow-sna shadow enhancer. Sequence of *rho*NEE is the same as one used in the previous study (3).

pbphi-DSCP$_{mMTE}$-MS2-yellow-rhoNEE

A DNA fragment containing *rho*NEE was inserted between HindIII and NheI sites of pbphi-DSCP$_{mMTE}$-MS2-yellow-sna shadow enhancer. Sequence of *rho*NEE is the same

as one used in the previous study (3).

pbphi-DSCP$_{mDPE}$-MS2-yellow-rhoNEE

A DNA fragment containing *rho*NEE was inserted between HindIII and NheI sites of pbphi-DSCP$_{mDPE}$-MS2-yellow-sna shadow enhancer. Sequence of *rho*NEE is the same as one used in the previous study (3).

DSCP$_{WT}$-MS2-yellow-gypsy-sna shadow enhancer

A DNA fragment containing *gypsy* insulator sequence was amplified using primers (5′-TTA AAA AGC TTC TGG CCA CGT AAT AAG-3′) and (5′-ACA TGA AGC TTG TTG TTG GTT GGC ACA CCA C-3′) and digested with HindIII. The resulting fragment was inserted into HindIII site of pbphi-DSCP-MS2-yellow-sna shadow enhancer (1). Sequence of *gypsy* insulator is the same as one used in the previous study (3).

DSCP$_{WT}$-MS2-yellow No enhancer

Two DNA oligos (5′-AGC TCC TCG TAG TTA AAT TCG GAG-3′) and (5′-CTA GCT CCG AAT TTA ACT ACG AGG-3′) were annealed and inserted between HindIII and NheI sites of pbphi-DSCP-MS2-yellow-sna shadow enhancer (1) to remove enhancer sequence.

DSCP$_{WT}$-MS2-yellow-IAB5 enhancer

A DNA fragment containing IAB5 enhancer was inserted between HindIII and NheI sites of pbphi-DSCP-MS2-yellow-sna shadow enhancer (1). Sequence of IAB5 enhancer is the same as one used in the previous study (3).

DSCP$_{mTATA}$-MS2-yellow-IAB5 enhancer

A DNA fragment containing IAB5 enhancer was inserted between HindIII and NheI sites of pbphi-DSCP$_{mTATA}$-MS2-yellow-sna shadow enhancer. Sequence of IAB5 enhancer is the same as one used in the previous study (3).

DSCP$_{mDPE}$-MS2-yellow-IAB5 enhancer

A DNA fragment containing IAB5 enhancer was inserted between HindIII and NheI sites of pbphi-DSCP$_{mDPE}$-MS2-yellow-sna shadow enhancer. Sequence of IAB5 enhancer is the same as one used in the previous study (3).

snaPr$_{WT}$-MS2-yellow-sna shadow enhancer

A DNA fragment containing WT *sna* core promoter sequence was amplified using primers (5′-TTT AAG CGG CCG CGA CAG CGG CGT CGG CAG AGG CGC-3′) and (5′-TTA AAG GAT CCT GGT TGC GTT CTC AAC GAG AGC TG-3′) and digested with NotI and BamHI. The resulting fragment was inserted between NotI and BamHI sites of pbphi-DSCP-MS2-yellow-sna shadow enhancer (1).

snaPr$_{mTATA}$-MS2-yellow- sna shadow enhancer

Two DNA oligos (5′-ACA TGA AGC TTG ACA GCG GCG TCG GCA GAG GCG CAG AGT TCC GGG GCG CCC CGA GCG TGC TCG ACT GTT GAC CT-3′) and (5′-AAT TTA GAT CTT GGT TGC GTT CTC AAC GAG AGC TGA GGT GGC TGT GAC AGG TCA ACA GTC GAG CAC GCT-3′) were annealed and blunt-ended by PCR. The resulting fragment was inserted between HindIII and BglII sites of pbphi plasmid after digestion with corresponding restriction enzymes. Subsequently, a DNA fragment containing mTATA *sna* core promoter sequence was amplified from the plasmid using primers (5′-TTT AAG CGG CCG CGA CAG CGG CGT CGG CAG AGG CGC-3′) and (5′-TTA AAG GAT CCT GGT TGC GTT CTC AAC GAG AGC TG-3′) and digested with NotI and BamHI. The resulting fragment was inserted between NotI and BamHI sites of pbphi-DSCP-MS2-yellow-sna shadow enhancer (1).

DSCP$_{3xZelda/mTATA}$-MS2-yellow-sna shadow enhancer

Two DNA oligos (5′-TTA AAG CGG CCG CCA GGT AGC AGG TAG CTC GAG CAG GTA GCC CGG GGA TCG AGC GCA GCG GGC GCC CCG GGC GCG GGG TGG CTG AGA GCA TCA GTT GTG AAT GAA TGT TCG AGC CGA GC-3′) and (5′-ACA TGG GAT CCG TTT GGT ATG CGT CTT GTG ATT CAA AGT TGG CTT ATT CAA AGG ATA TTA ACG AAG GCA GCG GCA CGT CTG CTC GGC TCG AAC ATT CAT T-3′) were annealed and blunt-ended by PCR. Resulting DNA fragment was inserted between NotI and BamHI sites of pbphi-DSCP-MS2-yellow-sna shadow enhancer (1) after digestion with corresponding restriction enzymes.

linker-DSCP-MS2-yellow

A DNA fragment containing 1.5-kb linker was amplified from *lacZ* sequence using primers (5′-TTT AAG CGG CCG CCC AAG ATC TCG CCT TGC AGC ACA TCC CCC-3′) and (5′-TTA AAC TCG AGC GAA AGC CAT TTT TTG ATG GAC C-3′) and digested with NotI and XhoI. The resulting fragment was inserted between NotI and XhoI sites of pbphi-DSCP-MS2-yellow (1).

rhoPr$_{WT}$-MS2-yellow-sna shadow enhancer

Two DNA oligos (5′-TTT AAG CGG CCG CTG GCT CTC TTA TAC TGC ACC GCA CAG CGA GCA CTT ATA AAG GCC GGC TGC CTT GCC TTT CGA AAG TCA GTT GCG TGC GA-3′) and (5′-TTA AAG GAT CCG TTT CAA TTT CAG TTT CGA GTC GCA GTC CGA CTT TCT CAG TTT GAT GAT CTC GCG CTC GCG GCT CGC ACG CAA CTG ACT-3′) were annealed and blunt-ended by PCR. Resulting DNA fragment was inserted between NotI and BamHI sites of pbphi-DSCP-MS2-yellow-sna shadow enhancer (1) after digestion with corresponding restriction enzymes.

rhoPr$_{mTATA}$-MS2-yellow-sna shadow enhancer

Two DNA oligos (5′-TTA AAG CGG CCG CTG GCT CTC TTA TAC TGC ACC GCA CAG CGA GCA CTG CGC CCG GCC GGC TGC CTT GCC TTT CGA AAG TCA GTT GCG TGC GAG CCG CGA GCG CG-3′) and (5′-TTA AAG GAT CCG TTT CAA TTT CAG TTT CGA GTC GCA GTC CGA CTT TCT CAG TTT GAT GAT CTC GCG CTC GCG GCT CGC ACG CAA CTG ACT-3′) were annealed and blunt-ended by PCR. Resulting DNA fragment was inserted between NotI and BamHI sites of pbphi-DSCP-MS2-yellow-sna shadow enhancer (1) after digestion with corresponding restriction enzymes.

rhoPr$_{mDPE}$-MS2-yellow-sna shadow enhancer

Two DNA oligos (5′-TTT AAG CGG CCG CTG GCT CTC TTA TAC TGC ACC GCA CAG CGA GCA CTT ATA AAG GCC GGC TGC CTT GCC TTT CGA AAG TCA GTT GCG TGC GA-3′) and (5′-ACA GTG GAT CCG TTT CAA TTT CAG TTT CGA GTC GCA GTC CGA CTT TCT CAG TTC ATG AGT CTC GCG CTC GCG GCT CGC ACG C-3′) were annealed and blunt-ended by PCR. Resulting DNA fragment was inserted between NotI and BamHI sites of pbphi-DSCP-MS2-yellow-sna shadow enhancer (1) after digestion with corresponding restriction enzymes.

rhoNEE-rhoPr$_{WT}$-MS2-yellow

A DNA fragment containing minimal *rho* core promoter sequence was amplified from rhoPr$_{WT}$-MS2-yellow-sna shadow enhancer using primers (5′-TTT AAC TCG AGT GCA CCG CAC AGC GAG CAC-3′) and (5′-TTA AAG GAT CCC GAC TTT CTC AGT TTG ATG A-3′), and digested with XhoI and BamHI. The resulting fragment was inserted between XhoI and BamHI sites of linker-DSCP-MS2-yellow. Subsequently, a DNA fragment containing *rho*NEE sequence was amplified from pbphi-DSCP$_{WT}$-MS2-yellow-rhoNEE using primers (5′-TTT AAG CGG CCG CTT CCT CTG CTC AAA ATC AAA

AT-3′) and (5′-TTT AAA GAT CTC CTC AGG TCG AGT TCC TCC A-3′), and digested with NotI and BglII. The resulting fragment was inserted between NotI and BglII sites of the plasmid.

rhoNEE-rho$_{mTATA}$-MS2-yellow

A DNA fragment containing minimal *rho* core promoter sequence was amplified from rhoPr$_{mTATA}$-MS2-yellow-sna shadow enhancer using primers (5′-TTT AAC TCG AGT GCA CCG CAC AGC GAG CAC-3′) and (5′-TTA AAG GAT CCC GAC TTT CTC AGT TTG ATG A-3′), and digested with XhoI and BamHI. The resulting fragment was inserted between XhoI and BamHI sites of linker-DSCP-MS2-yellow. Subsequently, a DNA fragment containing *rho*NEE sequence was amplified from pbphi-DSCP$_{WT}$-MS2-yellow-rhoNEE using primers (5′-TTT AAG CGG CCG CTT CCT CTG CTC AAA ATC AAA AT-3′) and (5′-TTT AAA GAT CTC CTC AGG TCG AGT TCC TCC A-3′), and digested with NotI and BglII. The resulting fragment was inserted between NotI and BglII sites of the plasmid.

rhoNEE-rhoPr$_{mDPE}$-MS2-yellow

A DNA fragment containing minimal *rho* core promoter sequence was amplified from rhoPr$_{mDPE}$-MS2-yellow-sna shadow enhancer using primers (5′-TTT AAC TCG AGT GCA CCG CAC AGC GAG CAC-3′) and (5′-TTA AAG GAT CCC GAC TTT CTC AGT TCA TGA G-3′), and digested with XhoI and BamHI. The resulting fragment was inserted between XhoI and BamHI sites of linker-DSCP-MS2-yellow. Subsequently, a DNA fragment containing *rho*NEE sequence was amplified from pbphi-DSCP$_{WT}$-MS2-yellow-rhoNEE using primers (5′-TTT AAG CGG CCG CTT CCT CTG CTC AAA ATC AAA AT-3′) and (5′-TTT AAA GAT CTC CTC AGG TCG AGT TCC TCC A-3′), and digested with NotI and BglII. The resulting fragment was inserted between NotI and BglII sites of the plasmid.

pBS-attP-dsRed-SV40

Two DNA oligos (5′-TCG ACA GTT CTA GAC CCC CAA CTG AGA GAA CTC AAA GGT TAC CCC AGT TGG GGG AAT TCA TCG ATA ACT TCG TAT AAT GTA TGC TAT ACG AAG TTA TG-3′) and (5′-CTA GCA TAA CTT CGT ATA GCA TAC ATT ATA CGA AGT TAT CGA TGA ATT CCC CCA ACT GGG GTA ACC TTT GAG TTC TCT CAG TTG GGG GTC TAG AAC TG-3′) were annealed and inserted into the modified version of pBS-3xP3-dsRed plasmid using SalI and NheI sites.

pCFD3-dU6-ftz-1

Two DNA oligos (5′-GTC GCT GCA AGG ACA TTT CGC CGG-3′) and (5′-AAA CCC GGC GAA ATG TCC TTG CAG-3′) were annealed and inserted into the pCFD3-dU6:3gRNA vector (addgene # 49410) using BbsI sites.

pCFD3-dU6-ftz-2

Two DNA oligos (5′-GTC GCA ATT TGT GAA GAA GAG TCT-3′) and (5′-AAA CAG ACT CTT CTT CAC AAA TTG-3′) were annealed and inserted into the pCFD3-dU6:3gRNA vector (addgene # 49410) using BbsI sites.

pBS-ftz 5′Arm-attP-dsRed-SV40-ftz 3′Arm

A DNA fragment containing 3′ homology arm of *ftz* was amplified from genomic DNA using primers (5′-TTT AAA CTA GTT CTT GGG CAT GCT GCA ATT TG-3′) and (5′-TTA AAG CGG CCG CGA AGG GTA GGA TAG AAT CTG-3′), and digested with SpeI and NotI. The resulting fragment was inserted between SpeI and NotI sites of pBS-attP-dsRed-SV40. Subsequently, a DNA fragment containing 5′ homology arm of *ftz* was amplified from genomic DNA using primers (5′-TTT AAG GTA CCA TGA AGA TCC TAC GCT GTG C-3′) and (5′-TTA AAG TCG ACG CGA AAT GTC CTT GCA GGC ACG-3′), and digested with KpnI and SalI. The resulting fragment was inserted between KpnI and SalI sites of the plasmid.

pbphi-ftz WT core promoter-ftz-HA-24xMS2-aTub 3′UTR

A DNA fragment containing *ftz* transcription unit fused with C-terminal HA-tag was amplified from genomic DNA using primers (5′-TTT AAG GAT CCA CTA GTA TGG CCA CCA CAA ACA GCC AGA GC-3′) and (5′-TTA AAG GAT CCT CAT GCA TAA TCC GGA ACA TCA TAC GGA TAA GAC AGA TGG TAG AGG TCC TGT GG-3′), and digested with BamHI. The resulting fragment was inserted between BglII and BamHI sites of the pbphi-hbP2 promoter-lacZ-24xMS2-αTub 3′UTR (modified version of pbphi-hbP2 promoter-lacZ-24xPP7-αTub 3′UTR described in (4)). Subsequently, a DNA fragment containing native *ftz* core promoter sequence was amplified from genomic DNA using primers (5′-TTT AAA AGC TTT GTC ATG CGC AGG GAT ATT TAT GCG-3′) and (5′-TTA AAA CTA GTA TCG GAT GTG TAT TGC TAG ATT TC-3′), and digested with HindIII and SpeI. The resulting fragment was inserted between HindIII and SpeI sites of the plasmid.

pbphi-ftz mTATA core promoter-ftz-HA-24xMS2-aTub 3′UTR

Two DNA oligos (5´-ACT ATA AGC TTT GTC ATG CGC AGG GAT ATT TAT GCG CTA TAA CGC CGA GCG TGT GCC GAG GGC TCT CTG ATT TTG CGC GCC CCG CAG GAT CTG CCG CAG G-3´) and (5´-AAT CCA CTA GTA TCG GAT GTG TAT TGC TAG ATT TCT TCT CTA ACT CTG CGA TGT GCA CGC AAC GCT GGT GAG TTT GCG AAT GAG CTG GTC CTG CGG CAG ATC CTG CGG G-3´) were annealed and blunt-ended by PCR. The resulting fragment was inserted between HindIII and SpeI sites of pbphi-ftz WT core promoter-ftz-HA-24xMS2-aTub 3´UTR after digestion with corresponding restriction enzymes.

pbphi-ftz mDPE core promoter-ftz-HA-24xMS2-aTub 3´UTR

Two DNA oligos (5´-ACA TGA AGC TTT GTC ATG CGC AGG GAT ATT TAT GCG CTA TAA CGC CGA GCG TGT GCC GAG GGC TCT CTG ATT TTG CTA TAT ATG CAG GAT CTG CCG CAG G-3´) and (5´-AAT CCA CTA GTA TCG GAT GTG TAT TGC TAG ATT TCT TCT CTA ACT CTG CAT GAG GCA CGC AAC GCT GGT GAG TTT GCG AAT GAG CTG GTC CTG CGG CAG ATC CTG CAT A-3´) were annealed and blunt-ended by PCR. The resulting fragment was inserted between HindIII and SpeI sites of pbphi-ftz WT core promoter-ftz-HA-24xMS2-aTub 3´UTR after digestion with corresponding restriction enzymes.

Supplemental References

1. Yokoshi, M., Segawa, K. and Fukaya, T. (2020) Visualizing the Role of Boundary Elements in Enhancer-Promoter Communication. *Mol Cell*, **78**, 224-235 e225.
2. Lim, B., Heist, T., Levine, M. and Fukaya, T. (2018) Visualization of Transvection in Living *Drosophila* Embryos. *Mol Cell*, **70**, 287-296 e286.
3. Fukaya, T., Lim, B. and Levine, M. (2016) Enhancer Control of Transcriptional Bursting. *Cell*, **166**, 358-368.
4. Fukaya, T., Lim, B. and Levine, M. (2017) Rapid Rates of Pol II Elongation in the *Drosophila* Embryo. *Curr Biol*, **27**, 1387-1391.
